# Age‐related telomere attrition in the human putamen

**DOI:** 10.1111/acel.13861

**Published:** 2023-05-02

**Authors:** Sebastian R. Schreglmann, Tomas Goncalves, Melissa Grant‐Peters, Demis A. Kia, Lilach Soreq, Mina Ryten, Nicholas W. Wood, Kailash P. Bhatia, Kazunori Tomita

**Affiliations:** ^1^ Queen Square Institute of Neurology University College London London UK; ^2^ Department of Neurology University Hospital Würzburg Würzburg Germany; ^3^ Chromosome Maintenance Group, UCL Cancer Institute University College London London UK; ^4^ Centre for Genome Engineering and Maintenance, College of Health, Medicine and Life Sciences Brunel University London London UK; ^5^ Genetics and Genomic Medicine, Great Ormond Street Institute of Child Health University College London London UK; ^6^ NIHR Great Ormond Street Hospital Biomedical Research Centre University College London London UK

**Keywords:** ageing, cerebellum, leukocyte, MM‐qPCR, putamen, spleen, substantia nigra, telomeres

## Abstract

Age is a major risk factor for neurodegenerative diseases. Shortening of leucocyte telomeres with advancing age, arguably a measure of “biological” age, is a known phenomenon and epidemiologically correlated with age‐related disease. The main mechanism of telomere shortening is cell division, rendering telomere length in post‐mitotic cells presumably stable. Longitudinal measurement of human brain telomere length is not feasible, and cross‐sectional cortical brain samples so far indicated no attrition with age. Hence, age‐related changes in telomere length in the brain and the association between telomere length and neurodegenerative diseases remain unknown. Here, we demonstrate that mean telomere length in the putamen, a part of the basal ganglia, physiologically shortens with age, like leukocyte telomeres. This was achieved by using matched brain and leukocyte‐rich spleen samples from 98 post‐mortem healthy human donors. Using spleen telomeres as a reference, we further found that mean telomere length was brain region‐specific, as telomeres in the putamen were significantly shorter than in the cerebellum. Expression analyses of genes involved in telomere length regulation and oxidative phosphorylation revealed that both region‐ and age‐dependent expression pattern corresponded with region‐dependent telomere length dynamics. Collectively, our results indicate that mean telomere length in the human putamen physiologically shortens with advancing age and that both local and temporal gene expression dynamics correlate with this, pointing at a potential mechanism for the selective, age‐related vulnerability of the nigro‐striatal network.

## Graphical abstract

Spleen (Leukocyte) mean telomere length correlates with mean telomere length in the putamen. Spleen can be used as the corresponding mean telomere length for biological age in post‐mortem samples.

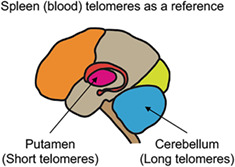

**Abbreviations**
CoV: coefficient of variation
CRBL: cerebellum
CWM: cerebral white matter
EWCE: expression‐weighted cell‐type enrichment
FCTX: frontal cortex
GWAS: Genome‐wide association studies
OCTX: occipital cortex
PMI: post‐mortem interval
PUTM: putamen
SNPs: single nucleotide polymorphisms
SPL: spleen
TRF: Telomere restriction fragment

## 1 INTRODUCTION

Telomeres are evolutionarily conserved repetitive hexanucleotide complexes at the ends of linear, eukaryotic chromosomes. They play an essential part in genomic stability by preventing end‐to‐end chromosome fusion (Shay & Wright, 2019). However, telomeres shorten with each cell division due to the “end‐replication problem” (Hug & Lingner, 2006). To prevent the loss of the protective telomere structure, shortened telomeres elicit DNA damage checkpoints that arrest the cell cycle, leading to cellular senescence or apoptosis (Artandi & Attardi, 2005). Therefore, mean telomere length defines cellular age and tissue regenerative capacity.

Our telomere length is largely defined during embryonic development (Daniali et al., 2013). The set length of telomeres is heritable and highly variable at birth, but typically averages over 10 kb in leukocytes (Broer et al., 2013; Codd et al., 2022; Holohan et al., 2015). Genome‐wide association studies (GWAS) have identified a number of single nucleotide polymorphisms (SNPs), genetically predicting leukocyte telomere length (Codd et al., 2013, 2021; Levy et al., 2010; Li et al., 2020). The strongest determinant of leukocyte telomere length is age, demonstrated by longitudinal studies (Benetos et al., 2013; Codd et al., 2022; Müezzinler et al., 2013). While highly dividing tissues such as blood, skin and gut typically have shorter telomeres than other tissues, telomeres in proliferative as well as minimally proliferative tissue shorten at a similar rate with age (Daniali et al., 2013). A large cohort study demonstrated a negative correlation between age and mean telomere length across most non‐germline tissue types, being strongest in tissue with shorter absolute telomere length (Demanelis et al., 2020). In addition to age, several environmental and lifestyle factors, such as smoking, BMI, physical activity and vitamin D deficiency (Hecker et al., 2021) and even paternal smoking status (Andreu‐Sánchez et al., 2022), influence telomere length. Collectively, on top of age, these genetic, epigenetic and environmental effects make telomere length variable and difficult to compare between individuals.

The importance of telomere length in disease predominantly manifests in short‐telomere‐associated syndromes, called telomere biology disorders or telomeropathies (Bertuch, 2016; Kam et al., 2021; Savage, 2018). Several monogenic conditions specifically illustrate the connection between short telomeres and premature ageing (Kam et al., 2021). Telomere shortening below a critical level leads to cellular replicative senescence, disturbed tissue renewal and malfunction (Baker et al., 2011; Chinta et al., 2018). Thus, mean telomere length has been proposed to be a measurable unit of biological age.

A plethora of epidemiological studies describe a correlation between leukocyte mean telomere length and diseases ranging from cardiovascular and degenerative diseases to many types of cancers (Barrett et al., 2015). A growing body of evidence also highlights a similar association between neurodegenerative (Eitan et al., 2014; Forero et al., 2016; Honig et al., 2012; Liu et al., 2016) and mental disorders (Huang et al., 2018; Powell et al., 2018; Vakonaki et al., 2018), as well as cognitive function (Hägg et al., 2017) and brain atrophy patterns (Jacobs et al., 2014; King et al., 2014). However, the physiological connections between such brain disorders and leukocyte mean telomere length remain elusive. Neurons are generally considered post‐mitotic or of slow renewal throughout adulthood (Kempermann et al., 2018), rendering neuronal telomere length presumably stable. Direct assessments of telomere length in human brain samples are relatively scarce (Allsopp et al., 1995; Nakamura et al., 2007; Takubo et al., 2002; Tomita et al., 2018) and longitudinal measurements with advancing age not feasible. Parkinson's disease risk is most strongly associated with advancing age (Dorsey et al., 2018; Pringsheim et al., 2014), and its pathophysiology pre‐eminently affects the substantia nigra and striatum, forming the nigro‐striatal network. The dynamics of telomere length in subcortical nuclei, like the striatum, have not been studied so far, and knowledge on telomere length between different brain regions is limited (Mamdani et al., 2015). Finally, the association of mean telomere length between the striatum and leukocytes has not been established.

In this work, based on matched spleen and brain post‐mortem samples from healthy individuals across the normal adult life span, we examined the physiological regional and temporal dynamics of human brain mean telomere length. By using spleen, rich in blood cells, as a reference for individual body telomere length, we demonstrate an age‐related telomere attrition in the putamen, an integral part of the basal ganglia and nigro‐striatal network as well as brain region‐specific differences in telomere length. Using available gene expression data, we identified the expression patterns of genes involved in telomere length regulation as a potential reason for physiological age‐ and region‐specific mean telomere length differences in the human brain.

## 2 RESULTS

### 2.1 Age‐related telomere attrition in human putamen

We determined telomere length in putamen and spleen (as a measure of the individual's biological age) from all *n* = 98 donors (sample group “putamen”)—in *n* = 10 of these we also determined telomere length in “supratentorial” samples (Tables 1, S1). The cause of sudden death was cardiac (*n* = 70 ischaemic; *n* = 4 structural), significant psychiatric comorbidity (*n* = 6 suicide; *n* = 4 intoxication), ruptured aortic aneurysm (*n* = 3), pulmonary embolism (*n* = 4), unascertained (*n* = 2), cerebral haemorrhage, asthma, diabetic coma, road traffic accident and peritonitis (*n* = 1 each). Due to tissue availability and demographic distribution, midbrain and substantia nigra samples were included from additional *n* = 9 “midbrain” sample donors (Table S2). Sample groups did not differ significantly in age (Welch's ANOVA test for unequal variance; *p* = 0.07) or pH (*p* = 0.16), while post‐mortem interval (PMI) was significantly longer in “midbrain” samples (*p* = 0.002).

**TABLE 1:** Donor demographic and tissue sample details of the collections used for telomere length quantification.

Tissue collection	“Putamen”	“Supratentorial”	“Midbrain”	
Region	SPL, PUTM (*n* = 98 each)	SPL, CWM, CRBL, FCTX, OCTX, PUTM (*n* = 10 each)	SPL, CRBL, SN, MB (*n* = 9 each)	
Age (mean ± SD; years)	52.0 ± 13.4	55.3 ± 1.95	53.4 ± 3.78	*p* = 0.07
Age range	20–79	53–58	47–58	
Sex (m/f; % male)	80/18; 82%	10/0; 100%	9/0; 100%	*p* = 0.13
pH	6.28 ± 0.24	6.36 ± 0.11	6.28 ± 0.09	*p* = 0.16
PMI	58.7 ± 22.1	51.3 ± 11.0	88.8 ± 20.8	*p* = 0.0009, # *p* = 0.005, $ *p* = 0.001

Abbreviations: CWM, cerebral white matter from Brodmann Area 39 & 40; CRBL, cerebellum, FCTX, frontal cortex; OCTX, occipital cortex; PUTM, putamen, SPL, spleen; PMI, post‐mortem interval; Welch's ANOVA test for unequal variance; comparison midbrain—putamen (#), comparison midbrain—supratentorial ($).

**FIGURE 1:**
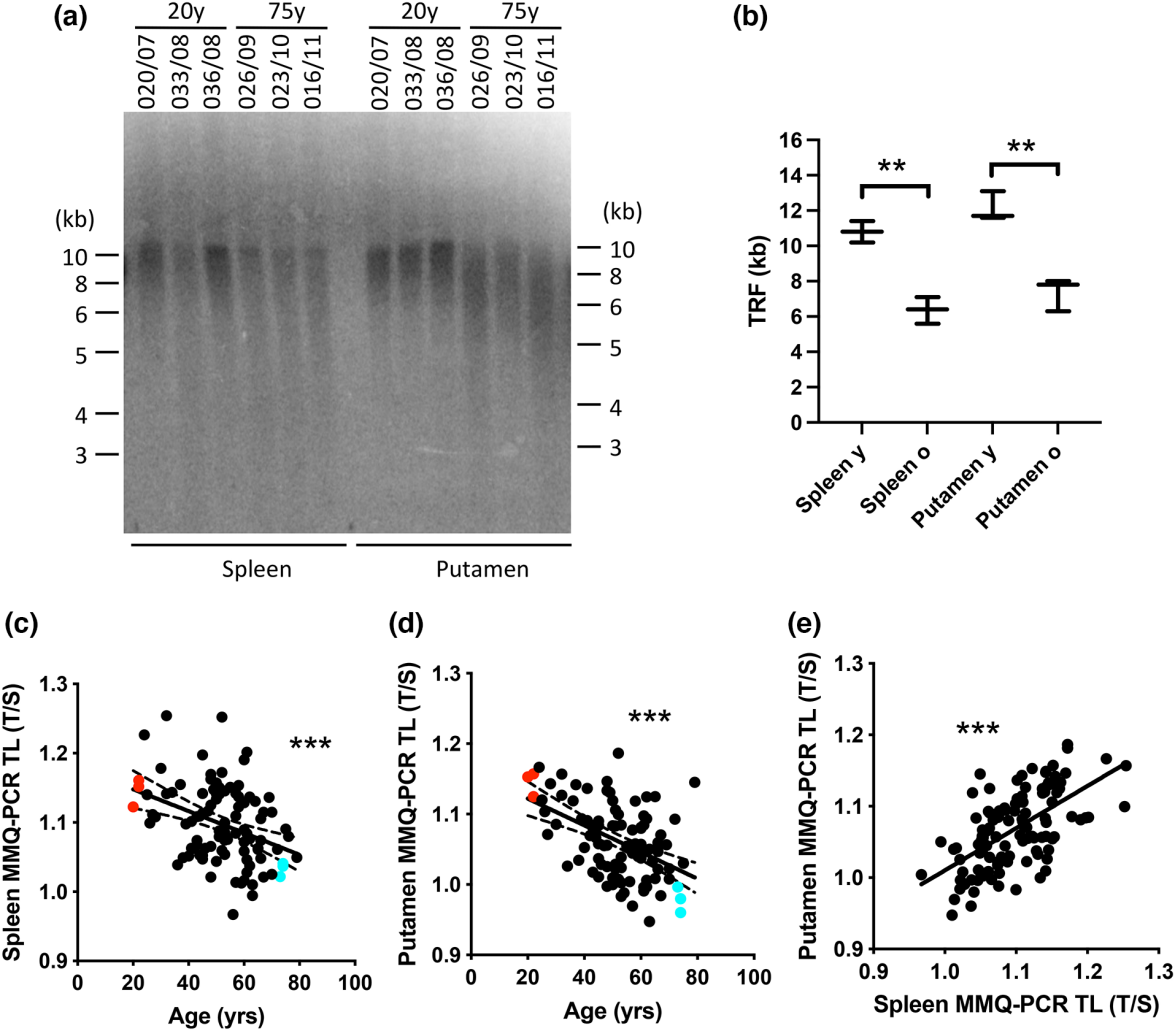
Temporal evolution of human putamen and spleen telomere length. (a–d) Telomere length was measured using (a, b) Telomere Restriction Fragment (TRF) Southern blot analysis and (c, d) MM‐qPCR. Absolute telomere length (a) was measured by using DNA ladder (kb), and compared between groups by median length quantification (b), showing significantly longer TL in young vs. aged spleen (*p* = 0.001) and putamen (*p* = 0.002) samples from corresponding sample pairs (*n* = 6 each, donor code is indicated); unpaired two‐tailed t test. Relative telomere length differences were assessed by MM‐qPCR, showing a negative correlation between age and relative telomere in both (c) spleen (Pearson correlation *r* = −0.378, *p* = 0.0001) and (d) putamen (Pearson correlation *r* = −0.48; *p* < 0.0001). Red and turquoise dots correspond to young (20 years old) and aged donors (75 years old) used in the TRF analysis (a, b), respectively. * indicates *p* < 0.05, ** indicates *p* < 0.005, *** indicates *p* < 0.0005 across the figure. (e) Graph shows a significant positive correlation between spleen (c) and putamen (d) telomere length (*n* = 98; Pearson correlation *r* = 0.6007, *p* < 0.0001).

Quantitative analysis using telomere restriction fragment (TRF) Southern blot comparison showed significantly longer telomeres in young (*n* = 3; 21.3 ± 0.9 years of age) compared with aged (*n* = 3; 73.7 ± 0.5 years of age) donors in both putamen (PUTM) and corresponding spleen (SPL) samples (Figures 1a,b, S1A). The distribution of short TRFs (minimal length and the 1st quartile) was proportional to the median telomere length (Figure S1B). This observation was expanded by showing a negative correlation of SPL and PUTM telomere length with advancing age in *n* = 98 corresponding sample sets from a normal age distribution as assessed using monochrome multiplex‐qPCR (MM‐qPCR) (Figure 1c–e). The coefficient of variation (CoV), calculated for repeated measurements across all tissue samples using MM‐qPCR, was 2.12% (*n* = 846), comparing well with previous studies (Balogh et al., 2020; Cawthon, 2009; Goncalves et al., 2020). The reliability and accuracy of MM‐qPCR were achieved by normalising with the reference cell line HEK293T as we described before (Goncalves et al., 2020). We confirmed that the mean telomere length, determined by the relative T/S in MM‐qPCR, correlated well with its minimum and 1st quartile (*r* ^2^ > 0.95), albeit less so with the median TRF and its first quartile (*r* ^2^ = 0.90 and *r* ^2^ = 0.87, respectively) on Southern blot (Figure S1C). Our result indicated a negative correlation of SPL mean telomere length with advancing age (Figure 1c: Pearson's *r* = −0.378, *p* = 0.0001), recapturing telomere length of peripheral blood. Surprisingly, this negative correlation was also found for PUTM mean telomere length (Figure 1d: *r* = −0.48, *p* < 0.0001), which did not substantially change after the removal of *n* = 10 samples of subjects with significant psychiatric comorbidity (*r* = −0.381, *p* = 0.0002). There was also a significant positive correlation between SPL and PUTM mean telomere length in our MM‐qPCR analysis (Figure 1e: *r* = 0.6007, *p* < 0.0001). We further analysed separately by sex. Whereas the correlation was independent of sex in SPL (male: *n* = 80, *F* = 9.13, *p* = 0.0034; female: *n* = 18, *F* = 6.0, *p* = 0.025), in PUTM this did not reach significance in females (male: *F* = 27.97, *p* < 0.0001; female: *F* = 3.09, *p* = 0.09), presumably due to low number of female samples. Collectively, our comparative telomere length analysis between matched putamen and spleen (blood) samples indicated that telomeres in putamen shorten with age.

### 2.2 Telomere length is region‐specific in the human brain

**FIGURE 2:**
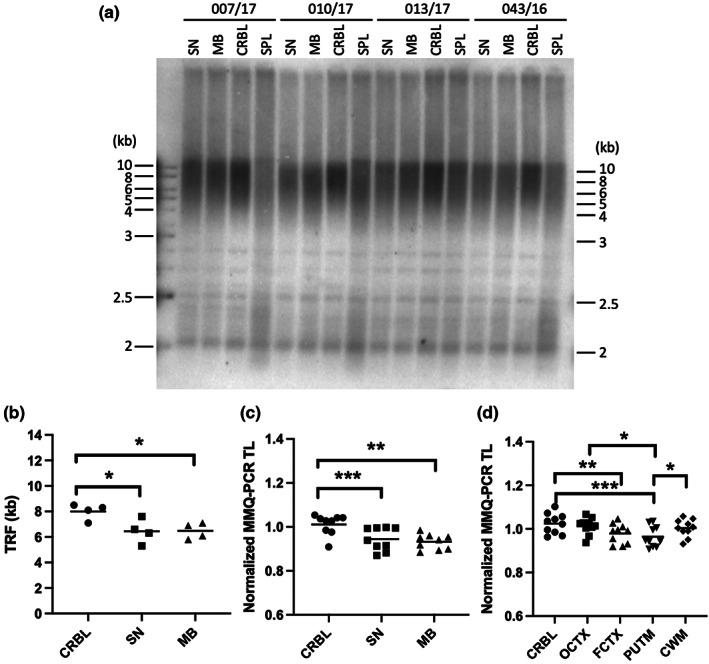
Human brain telomere length is region‐specific. (a) TRF Southern blot analysis of matched substantia nigra (SN), midbrain (MB), cerebellar cortex (CRBL) and spleen (SPL) samples from 4 donors (donor code is indicated). (b) Statistical analysis of median telomere length calculated from TRF (a) shows that CRBL telomeres were longer than SN and MB (repeated measures ANOVA *F* = 8.12; *p* = 0.04). (c, d) MM‐qPCR relative telomere length was normalised to matched SPL and indicated brain regions from midbrain and supratentorial samples were compared with CRBL. (c) Statistical analysis confirmed longer telomeres in CRBL than in SN or MB (*n* = 9; repeated measures ANOVA *F* = 19.22; *p* < 0.0001) and (d) CRBL than in PUTM (*n* = 10; repeated measures ANOVA *F* = 16.05; *p* < 0.0001). Tissue collection “midbrain” comprises of samples from donors 007/17, 010/17, 013/17, 032/16, 040/16, 042/16, 043/16, 048/16 and 051/16, “supratentorial” samples stem from donors 030/06, 034/06, 035/06, 026/07, 007/08, 018/09, 033/09, 041/10, 012/11 and 032/11. ANOVA post hoc test results are displayed with *p*‐values corrected for multiple comparison. Across the figure, * indicates *p* < 0.05, **p < 0.01 and ***p < 0.001.

To explore region‐specific telomere length dynamics, we next compared samples from corresponding brain regions. According to tissue availability, median telomere length of matched Substantia nigra (SN), midbrain (MB) and cerebellar cortex (CRBL) and SPL (*n* = 4, all male, mean age 52.0 ± 3.6 years) were measured by TRF Southern blot (Figure 2a). The average telomere length varied between donors (Figure S2A). In order to compare telomere length of brain tissues in a small cohort, we used matched SPL median telomere length as a reference of inter‐individual variability and determined relative telomere length over the corresponding SPL, as the leukocyte telomere length represents measurable “biological” age (Figure 2b). Unlike previous reports (Mamdani et al., 2015), our results showed that MB and SN contains significantly shorter telomeres than CRBL. Using total *n* = 9 “midbrain” samples (Table 1), this observation was further assessed by relative MM‐qPCR telomere length measurements (Figure S2B). By normalising over SPL mean telomere length, we confirmed shortened telomeres in midbrain samples compared with the cerebellum (Figure 2c).

We next assessed mean telomere length between CRBL and supratentorial regions including occipital cortex (OCTX), frontal cortex (FCTX), PUTM and cerebral white matter (CWM) as well as SPL in “supratentorial” samples from *n* = 10 donors (see Table 1). Our relative MM‐qPCR telomere length analysis indicated telomeres to be longest in CRBL and shortest in PUTM after normalising to SPL mean telomere length (Figures 2d, S2C). Collectively, our data indicated that telomeres in the PUTM and SN/MB are shorter than in the CRBL.

### 2.3 Distinct expression of telomere‐related genes between the cerebellum and the nigro‐striatal system

**FIGURE 3:**
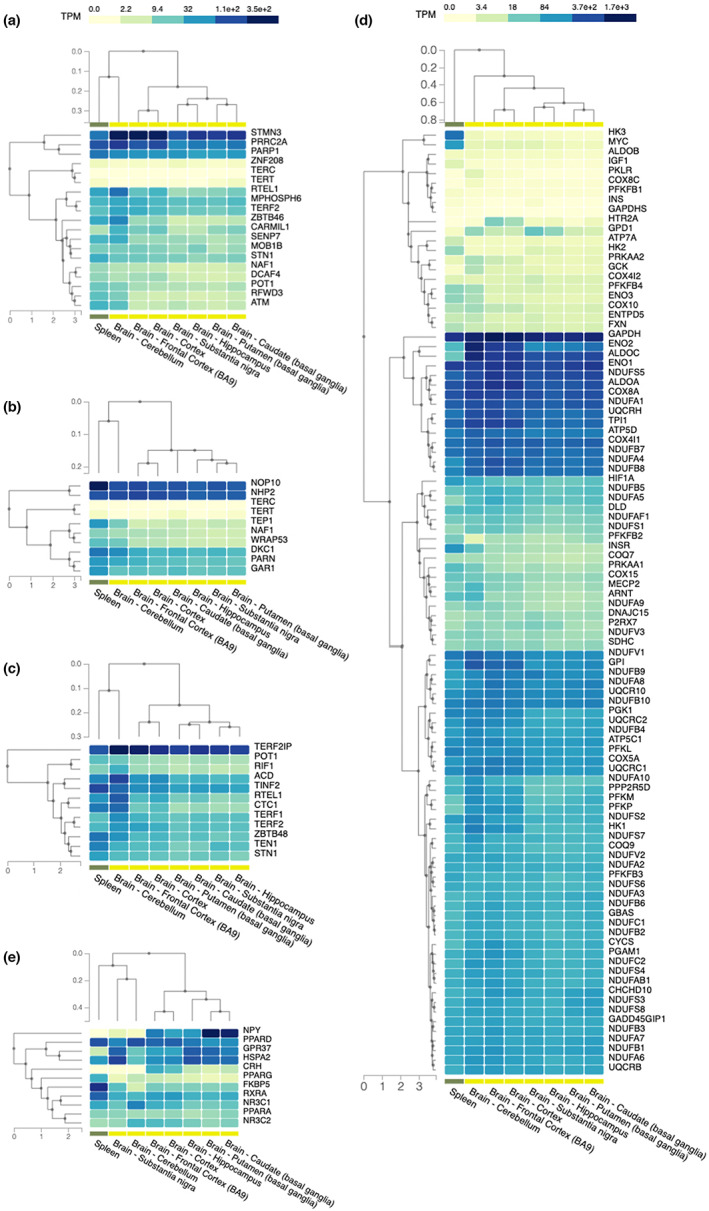
Region‐specific expression of genes involved in telomere length regulation. Expression levels of genes specific to (a) telomere length heritability, (b) telomerase biogenesis, (c) telomere maintenance, (d) oxidative phosphorylation and (e) stress response/neuroprotection across brain regions and spleen—as proxy to blood lymphocytes—are plotted as clustered heatmaps using hierarchical clustering. Gene regulation follows a region‐specific pattern with genes involved in TL inheritance, maintenance, telomerase biogenesis and oxidative phosphorylation showing relatively highest expression in cerebellum with putamen (and the nigro‐striatal system including substantia nigra) as well as the hippocampus showing lowest expression levels (a–d). Regulation of stress response/neuroprotection genes cluster slightly different, with the substantia nigra clustering closer to the cerebellum than putamen (e). Given unit: transcript per kilobase million (TPM).

To probe the underlying mechanism of telomere attrition and region‐specific mean telomere length in the brain, we explored the differences in transcription patterns among the brain regions using the Genotype‐Tissue expression (GTEx) project dataset (Lonsdale et al., 2013). We focused and compared the region‐specific expression of genes involved in telomere length inheritance, telomerase biogenesis, telomere maintenance, oxidative phosphorylation and stress response/neuroprotection. Within the panel of telomere length inheritance genes, the expression frequently differed by a factor of magnitude between the studied brain regions (Figure 3a). Expression of the genes encoding the telomerase core components, *TERT* and *TERC*, were low or hardly detectable in any brain region. All genes related to telomere biology were expressed in a region‐dependent manner (Figure 3a–c). Interestingly, hierarchical clustering of gene expression trends arranged an anatomical order with the cerebellum on one and the nigro‐striatal system on the opposite end. In addition, the hippocampus tended to cluster more with the basal ganglia than cortical areas. Strikingly, a similar expression pattern was detected among genes involved in oxidative phosphorylation (Figure 3d). In the analysis on stress response/neuroprotection gene regulation, substantia nigra however clustered close to the cerebellum and far from the other parts of the nigro‐striatal network, deviating from the pattern observed in the previous gene categories (Figure 3e). Hence, among the studied samples, genes that regulate telomere length were controlled in a region‐dependent manner with expression patterns matching mean telomere length.

As neurons are more abundant in the cerebellum than putamen (Herculano‐Houzel, 2014), potentially explaining differences in gene expression levels, we aimed to explore whether telomere length‐regulating genes are expressed in a cell‐type‐dependent manner. To this end we applied the expression‐weighted cell‐type enrichment (EWCE) analysis. This revealed that neuronal cell types and oligodendrocytes had significantly enriched expression for genes involved in telomere length inheritance/maintenance/biogenesis, oxidative phosphorylation, and stress response when compared to analogous bootstrapped gene sets (Figure S3). Oligodendrocytes represent the majority of glial cells across the human brain (von Bartheld et al., 2016). Our data support cell‐type‐specific expression for these particular gene categories but did not differentiate two main cell types governing telomere length. Hence, cell‐type specificity appears not to contribute to regional gene expression differences.

### 2.4 Age‐related expression changes of telomere length‐regulating genes are brain region‐specific

**FIGURE 4:**
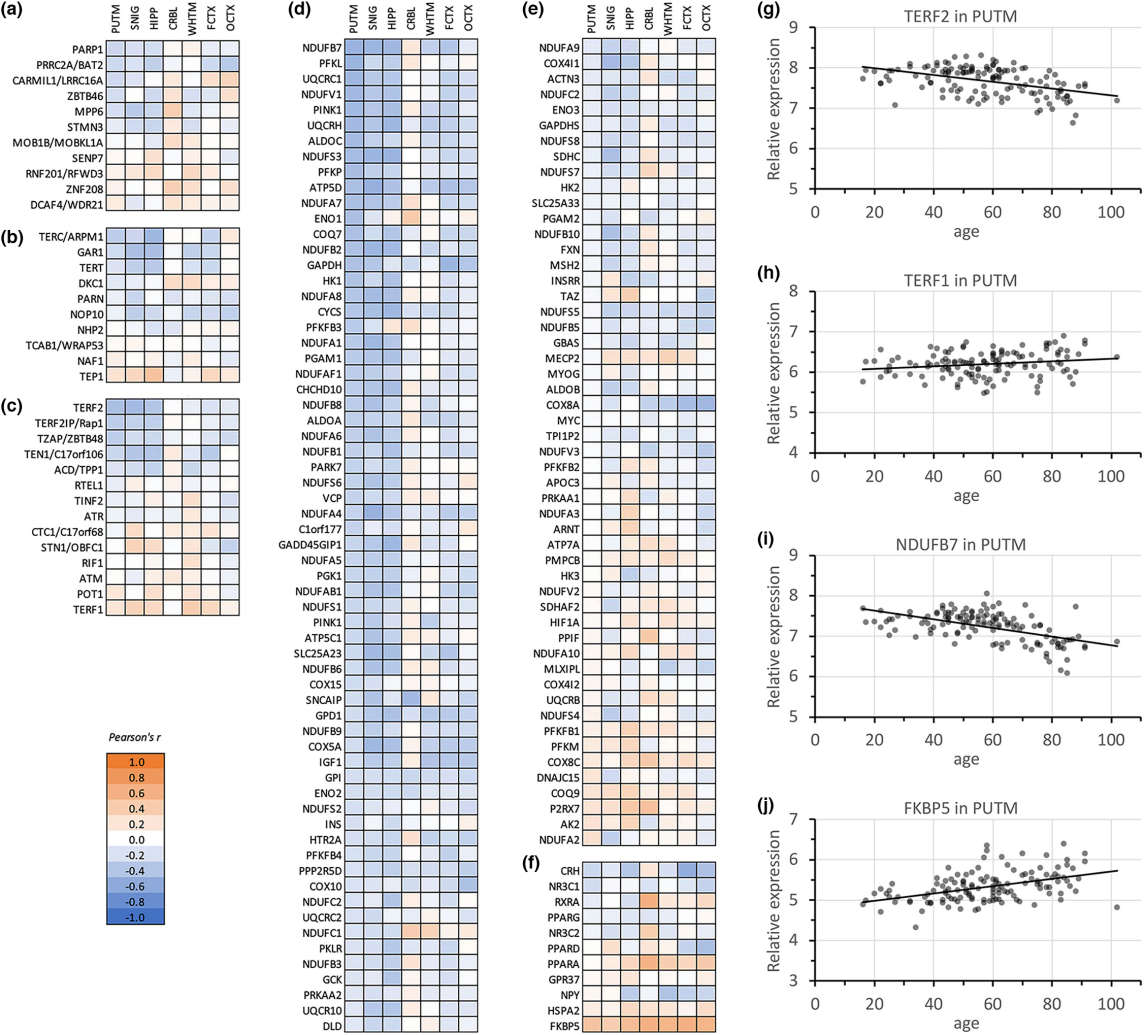
Age‐related changes in expression levels of genes involved in telomere length regulation and stress responses. (a–f) Heatmaps summarising the degree of linear correlation between gene expression level of telomere length‐regulating genes and age (Pearson correlation coefficient) across seven studied brain regions. Positive and negative correlation are indicated by warm and cold colours, respectively (numerical results in Table S3). Analysed gene categories are (a) telomere length inheritance, (b) telomere maintenance, (c) telomerase component/biogenesis, (d, e) oxidative phosphorylation and (f) stress/neuroprotection. PUTM (putamen, *n* = 129); SNIG (substantia nigra, *n* = 101); HIPP (hippocampus, *n* = 122); CRBL (cerebellum, *n* = 130); WHMX (intralobular white matter, *n* = 130); FCTX (frontal cortex, *n* = 127); OCTX (occipital cortex, *n* = 129). (g–j) Exemplary scatter plots show the linear correlation between age and gene expression level of either (g) TERF2, (h) TERF1, (i) NDUFB7 or (j) FKBP5 in the putamen.

Regional difference in the transcription of the telomere regulating genes prompted us to investigate whether similar changes are detectable with age. To this end, we analysed age‐related gene expression differences across brain regions from the UK Brain Expression Consortium (UKBEC) data. In this dataset, gene expression across 13 brain regions from 134 adult post‐mortem brains was quantified using DNA microarray, and relative expression was calculated using housekeeping genes (Trabzuni et al., 2011). For our analysis, we focused on the same gene categories and brain regions as above (Figures 2, 3). The degrees of correlation between gene expression level and age are summarised in a heatmap (Figure 4a–f, Table S3). Among analysed gene sets, interestingly the gene expression pattern again appeared similar between putamen, substantia nigra and hippocampus and clearly differed from other brain regions, in particularly for oxidative phosphorylation, telomere maintenance and telomerase biogenesis. In terms of telomere biology, our data identified strongest negative correlation with age for the expression of *TERF2* (Telomeric repeat‐binding factor 2) and *TERF2IP* (TERF2 interacting protein) in these three regions (Figure 4c,g). *TERF1* on the contrary, a gene encoding another telomeric repeat‐binding factor, was upregulated with age (Figure 4c,h). This was in striking difference to the cerebellum, harbouring longer telomeres, where we did not observe similar expression changes. Overall, the strongest gene expression changes were identified for oxidative phosphorylation‐related genes, which again showed a clear region‐specific pattern with very similar changes in the nigro‐striatal system and hippocampus and strikingly different findings particularly in the cerebellum (Figure 4d,e). *NDUFB7* and other *NDUF* genes that encode a subunit of the mitochondrial NADH–ubiquinone oxidoreductase (complex 1) were notably downregulated with age except in cerebellum (Figure 4d,e,i), implying potential age‐related reduction of mitochondrial activity in these brain regions. The only gene showing clear upregulation with age across all analysed seven regions was *FKBP5* (Figure 4f,j). Collectively, gene expression changes with age appeared in a region‐specific manner with predominant downregulation of telomeric genes (especially *TERF2* and *TERF2IP*) and genes regulating oxidative phosphorylation in the nigro‐striatal system and hippocampus.

Age‐related downregulation of *TERF2* in the putamen prompted us to assess whether brain region‐specific telomere length is associated with the level of *TERF2* expression. Using our telomere length analysis and sequencing‐based GTEx RNA expression data from a large number of samples (*n* = 114–209, depending on region) from different brain regions, we established a correlation between region‐specific mean telomere length and *TERF2* expression (Figure S4). However, this did not replicate when analysing telomere length and available gene expression from the much smaller set of 76 putaminal samples overlapping between our sample set and UKBEC DNA microarray data for *TERF2* expression for neither mean telomere length (Pearson's *r* = 0.171; *p* = 0.14) nor age (Pearson's *r* = −0.083, *p* = 0.48). In addition to the more accurate expression quantification by sequencing in GTEx, much lower sample numbers are a likely limitation for the analysis based on matched UKBEC samples.

Taking altogether, the putamen as part of the nigro‐striatal network, differs substantially from other human grey matter regions not only in terms of regional, but also temporal dynamics in the expression levels of genes involved in telomere length regulation.

## 3 DISCUSSION

Our results, based on relative (MM‐qPCR) and absolute (TRF Southern blot) telomere length measurements from unique, matched spleen and brain tissue samples from healthy humans without neurological or neurodegenerative disease, covering the normal adult life span between 20 and 79 years of age, are consistent and robust. With our novel approach to use matched spleen and putamen samples, we found a positive correlation between blood and putamen mean telomere length and demonstrated a physiological age‐related telomere attrition in the human putamen with advancing age, so far only reported for the human hippocampus (Demanelis et al., 2020). This is surprising as the putamen is rich in post‐mitotic neurons, generally thought to exhibit no relevant change in telomere length (Tomita et al., 2018). Our analyses further confirmed region‐specific mean telomere length with the longest telomeres in the cerebellum and the shortest in the nigro‐striatal system at the age of fiftieth. Our analysis of the region‐ and age‐specific transcriptomes elucidated that the transcriptional landscape of telomere‐related genes is distinct between the cerebellum and the nigro‐striatal system, suggesting the presence of physiological mechanisms that define regional brain telomere length regulation. Although further investigations are required, this study establishes age‐related telomere shortening in the human nigro‐striatal system and the likely underlying role of region‐ and age‐specific differences in gene regulation.

### 3.1 Limitations of brain telomere epidemiology and its developments

Previous reports did not document age‐related changes in telomere length during adult life from pooled cerebral (Allsopp et al., 1995) as well as occipital cortical (Nakamura et al., 2007; Takubo et al., 2002; Tomita et al., 2018) and subcortical samples (Mamdani et al., 2015). However, sample characteristics and involved methods might explain this:

The first issue is the number and age distribution of donors. For some studies, post‐mortem donors have been pooled from infancy and very late adulthood (50–100 years of age) into the same analyses, extrapolating results for much of the normal adult life span (Allsopp et al., 1995; Nakamura et al., 2007; Takubo et al., 2002). The foetal and infant period is a time of rapid neural growth and development (Paredes et al., 2016), including drastic changes in the degree of myelination (Deoni et al., 2011). Indeed, leukocyte telomeres rapidly shorten during childhood (Aubert & Lansdorp, 2008; Zeichner et al., 1999). Our present study provides an even sample distribution across the normal adult life span, especially covering young adult to mid‐aged adult groups.

The second issue is the variability of individual telomere length at birth due to genetic variability and parental inheritance (Demanelis et al., 2020; Holohan et al., 2015). Telomere length measured in cross‐sectional studies is the combination of telomere length at birth and age‐related attrition up until sample collection. Such bias can be minimised in a large population study but has not been accounted for in previous brain telomere studies (Allsopp et al., 1995; Mamdani et al., 2015; Nakamura et al., 2007; Takubo et al., 2002; Tomita et al., 2018). Importantly, telomere length correlates closely between different tissues of the same individual (Daniali et al., 2013; Dlouha et al., 2014). By exploiting the fact that leukocytes are the only longitudinally available source for age‐related telomere attrition in humans (Müezzinler et al., 2013), we used matched spleen as a reference for relative telomere length of the brain to provide convincing evidence for a true age‐related attrition of telomeres in the human putamen with age. This strategy also enabled us to compare relative telomere length of the brain regions in the same age group.

The third issue is the methodology and reliability of the telomere measurement assays. Our region‐specific telomere length measurements using both TRF and MM‐qPCR methods provided complementary results. But, our results did not recapture telomeres in the substantia nigra to be 10–20 times longer than in other brain regions, as reported previously (Mamdani et al., 2015). We speculate that this difference was due to the experimental setting of MM‐qPCR. Because this method uses qPCR for quantification of telomere repeats, the CoV among repeat experiments can be substantial (Cawthon, 2009). The large cohort (UK biobank) telomere length analysis elucidated the technical factors that influence CoV (Codd et al., 2022). We circumvent this by the use of a standard, well‐characterised HEK293 reference cell line, previously employed to the Flow‐qFISH method (Baerlocher et al., 2006), as a reference for each qPCR run, minimising variability.

### 3.2 How do telomeres in the brain shorten with age?

Chromosome replication is the major source of telomere attrition. Although controversial, low‐level mitotic activity is observed in a sub‐population of cells in the human striatum (Cipriani et al., 2018; Eriksson et al., 1998; Ernst et al., 2014; Sorrells et al., 2018; Spalding et al., 2013). Another human brain area with reported age‐related telomere length attrition is the hippocampus (Demanelis et al., 2020), where there is evidence for human adult neurogenesis up to the 10th decade of life (Moreno‐Jiménez et al., 2021). Nevertheless, an estimated annual striatal neuronal turnover rate of 2.5% during adulthood (Ernst et al., 2014) is unlikely to explain the rate of telomere shortening solely by the end‐replication problem.

Metabolic stress is another hypothetical reason: In the striatum, medium spiny neurons constitute 95% of striatal neurons with a known selective vulnerability to metabolic stress due to their excessive baseline energy expenditure to maintain cell membrane ion gradients (Mitchell et al., 1999). Indeed, we observed age‐related reduction in gene expression involved in oxidative phosphorylation, especially mitochondrial complex 1. Such chronic metabolic stresses accumulate reactive oxygen species (ROS) (Smeitink et al., 2004). ROS has been shown to lead to telomere attrition in vitro (Von Zglinicki, 2002), which is caused by ROS‐mediated telomeric DNA damage followed by chromosome replication and segregation (Barnes et al., 2019; Coluzzi et al., 2019). Whereas epidemiological studies indicated a correlation between leukocyte telomere length and perceived and chronic stresses that associated with ROS, there is still conflicting evidence in in vivo models and the molecular basis of telomere shortening by ROS remains to be fully understood (Boonekamp et al., 2017; Pineda‐Pampliega et al., 2020). Whether the cell cycle is required or not, the direct effects of ROS on telomere shortening in quiescent or slow‐renewing cells like neurons remain to be established in humans.

Alternatively, changes in the absolute number of, or the proportion between cell populations might explain the reduction in mean telomere length in the putamen. Previous studies did not find age‐related telomere attrition in human neuronal and glial cells across the adult age range in healthy controls but identified significantly shorter telomeres in glial cells compared with neurons (Tomita et al., 2018). Stereology studies consistently reported decreasing number of oligodendrocytes but not neurons with advancing age in the human neocortex (Fabricius et al., 2013; Pelvig et al., 2008), striatum and putamen (dos Santos‐Lobato et al., 2015; Selden et al., 1994). Similarly, changes in cell‐type‐specific gene expression did not indicate a decrease in neuronal markers with age (Soreq et al., 2017). Our EWCE analysis revealed significant enrichment for telomere length‐regulating genes in oligodendrocytes as well as all types of neurons, indicating that there is no difference in expression levels between these two main telomere length‐defining cell populations in the putamen. It therefore remains unknown whether the observed decrease in mean telomere length in the striatum is the result of a cell‐type‐specific or a more general, region‐specific effect on telomere length, as our experimental approach can not differentiate telomere attrition in neuronal and glial cells.

One limitation is that naturally most subjects included in this study died of underlying health conditions, which is an inherent limitation of human healthy control post‐mortem studies—in fact the majority of subjects died of cardiovascular disease. Although we can exclude the influence of neurological and neurodegenerative disorders by clinical information and neuropathological examination, as well as major psychiatric disorders by the above calculations on our findings, sample numbers do not allow to sufficiently correct for other factors possibly influencing mean telomere length (Hecker et al., 2021).

### 3.3 Age and region‐associated expression of genes regulating telomere length

Our analysis revealed an age‐associated attrition of telomeres in the putamen. Interestingly, a previous study evaluating mean telomere length over age showed a clear trend for age‐related attrition in the substantia nigra of healthy controls (Hudson et al., 2011). We hence note that telomere length dynamics in the nigro‐striatal system seem to differ substantially from previously studied brain regions that showed no attrition with age (Allsopp et al., 1995; Nakamura et al., 2007; Takubo et al., 2002; Tomita et al., 2018). We found that the genetic telomere length regulation was similar throughout the nigro‐striatal system and was different from regions with longer telomeres, such as the cerebellum.

Interestingly, our analyses also indicate that genes involved in stress response are regulated differently in the substantia nigra compared with the rest of the nigro‐striatal network. Different populations of neurons show various degrees of vulnerability to oxidative stress (González‐Rodríguez et al., 2019) and cerebellar granule cells and substantia nigra dopaminergic neurons have been reported to be particularly sensitive (Wang & Michaelis, 2010). This selective population‐specific vulnerability shared between substantia nigra and cerebellum is one possible explanation why these two brain regions—in contrast to all other gene categories analysed—cluster in the stress response/neuroprotection gene expression category. Together with the variable effect size of the respective gene variants (Codd et al., 2013; Li et al., 2020), this could explain the observed differences in region‐specific telomere length.

As with the regional telomere length difference found, our age‐related gene expression analyses identified a similar clustering of the nigro‐striatal system with the hippocampus and in striking difference to the cerebellum, suggesting that similar mechanisms could be connected to age‐related telomere shortening. Along several genes regulating oxidative phosphorylation, telomeric genes *TERF2* and *TERF2IP*, involved in forming the shelterin complex (Lim & Cech, 2021), showed decreased expression in SN and PUTM with age. Our results however do not unequivocally support a major role of *TERF2* expression on telomere length, as they did not replicate in all tested datasets. However, the relative low sample number and limited resolution of DNA microarray gene expression data are likely limitations. Further studies are needed to explore telomere length control specifically in post‐mitotic neurons.

Advancing age is a well‐established risk factor for neurodegenerative disease (Pringsheim et al., 2014), and the nigro‐striatal system is intricately tied to Parkinson's disease (PD) pathophysiology (Poewe et al., 2017). So far, impaired lysosomal, proteasomal and mitochondrial function, inflammation and oxidative stress have been suggested to be shared mechanisms in ageing and PD (Collier et al., 2017; Hudson et al., 2011; Reeve et al., 2014). Furthermore, both epidemiological (Forero et al., 2016) and the Mendelian randomisation‐based studies (Chen & Zhan, 2021) have not provided evidence of an influence of leukocyte telomere length on PD risk. Nevertheless, age‐related gene expression changes have been described predominantly to occur in the nigro‐striatal system and hippocampus—the two regions archetypically connected to the two most prevalent age‐related neurodegenerative diseases, namely Alzheimer's dementia and PD (Soreq et al., 2017).

The discovery of a physiological, age‐related telomere attrition in the putamen/the nigro‐striatal system, so far only observed in the hippocampus (Demanelis et al., 2020) but not in other human brain regions, might serve as a potential additional explanation for the close association between advancing age and PD as well as hippocampal‐related Alzheimer's dementia risk. Future studies both in the field of Parkinson's and Alzheimer's disease research should investigate how changes in telomere length with advancing age mechanistically relate to neurodegeneration.

## 4 EXPERIMENTAL PROCEDURES

### 4.1 Human post‐mortem brain samples

Tissue samples from control subjects without history or neuropathological features of a neurological or neurodegenerative disease were identified at the MRC Sudden Death Brain and Tissue Bank (Millar et al., 2007). All tissue handling was done according to local standardised procedures after ethical approval by the local Ethics board (NHS Lothian LREC 2003/8/37) and in accordance with the Declaration of Helsinki. Three collections of tissue samples were identified from *n* = 107 healthy donors across the adult age according to tissue availability: “putamen” (*n* = 98; 82% male; from PUTM), “supratentorial” (*n* = 10 male donors closely matched for age, brain tissue pH and post‐mortem delay; containing cerebral white matter (CWM), frontal cortex (FCTX) and occipital cortex (OCTX)) and “midbrain” (*n* = 9 closely matched male donors; containing substantia nigra pars compacta (SN) and adjacent dorsal MB). All three tissue collections included corresponding spleen samples, “supratentorial” and “midbrain” collections also included cerebellar cortex (CRBL) samples (see Tables 1, S1 and S2 for details). Samples were kept frozen throughout the dissection process under DNase/Rnase free conditions. DNA was extracted using LGC Genomics sbeadex livestock kit (LGC genomics, no. 44702). Quality and quantity of genomic DNA were assessed by Ethidium bromide staining followed by electrophoresis and Qubit fluorometer (Invitrogen/thermo fisher scientific) measurements.

### 4.2 Telomere restriction fragment (TRF) analysis

TRF analysis using Southern blot was performed as described with minor modification (Goncalves et al., 2020). Briefly, 2 μg of genomic DNA was digested with *Rsa*I and *Hin*fII at final volume 30 μL in accompanying Smart Cut buffer (NEB) at 37°C overnight. Digested genomic DNA was separated in 30‐cm long 0.8% agarose gel and transferred to a nylon membrane (Hybond N‐; GE Healthcare) via capillary action after treating a gel with 1 N sodium chloride and alkaline solution (sodium hydroxide). DNA on the membrane was crosslinked by UV and hybridised with a ^32^P‐labelled human telomeric DNA fragment probe. Telomere length was analysed using *WALTER* (Lyčka et al., 2021).

### 4.3 Monochrome multiplex quantitative PCR (MM‐qPCR)

MM‐qPCR analysis was performed using genomic DNA samples at 5 ng/μl as described (Goncalves et al., 2020). Genomic DNA from HEK293K cell line (ATCC® CRL‐3216™) was used as a reference and standard, and Ct values were calculated from triplicate measures using Quant Studio 5 (Fisher Scientific). Primer sequence for telomeric DNA and albumin are described in (Cawthon, 2009). qPCR results with skewed Ct values (CoV > 1%) were rejected. The mean R^2^ of telomere and albumin measurements were 0.9968 and 0.9970, correspondingly. T/S ratio was then calculated as T (the number of nanograms of the standard DNA that matches the experimental sample for copy number of the telomere template) divided by S (the number of nanograms of the standard DNA that matches the experimental sample for copy number of albumin). Telomere template copy number is presented as fold enrichment against the reference HEK293T standard in the same reaction plate to minimise day‐to‐day variation of qPCR. MM‐qPCR triplicate measurements were done at least three times per sample. There was good correlation between repeat measurements in SPL (*R* ^2^ = 0.81, *p* < 0.0001), PUTM (*R* ^2^ = 0.87, *p* < 0.0001), as well as all region‐matched brain samples (*R* ^2^ = 0.68, *p* < 0.001). Statistical (correlation analysis; unpaired, two‐tailed student t test; Welch's ANOVA for unequal variance with Dunnett's multiple comparison and one‐way ANOVA with Tukey's multiple comparisons) analysis were done using GraphPad Prism v8.0.

### 4.4 Gene expression analysis

Brain region‐specific expression of genes identified to be associated with leukocyte telomere length inheritance (*PARP1, SENP7, TERC, MOB1B, NAF1, TERT, PRRC2A, CARMIL1, POT1, STN1, ATM, DCAF4, TERF2, MPHOSPH6, RFWD3, ZNF208, ZBTB46, STMN3 and RTEL1*), the gene list taken from (Li et al., 2020), telomerase biogenesis (*TERT, TEP1, TERC/TR, WDR79/WRAP53, DKC1, NOP10, GAR1, NHP2, NAF1 and PARN*), telomere maintenance (*POT1, TERF1, TERF2, ACD, TINF2, TERF2IP, CTC1, STN1, TEN1, RIF1, RTEL1 and TZAP/ZBTB48*), the gene list taken from (Lim & Cech, 2021), oxidative phosphorylation (*ENO1, ENO2, ENO3, ENTPD5, GAPDHS, GAPDH, GCK, GPD1, GPI, HIF1A, HK1, HK2, HK3, HTR2A, IGF1, INSR, MYC, P2RX7, PFKFB1, PFKFB2, PFKFB3, PFKFB4, PFKL, PFKM, PFKP, PGAM1, PGAM2, PGK1, PKLR, PPP2R5D, PRKAA1, PRKAA2, TPI1, INS, ALDOA, ALDOB, ALDOC, ARNT, NDUFA10, NDUFA1, NDUFA2, NDUFA3, NDUFA4, NDUFA5, NDUFA6, NDUFA7, NDUFA8, NDUFA9, NDUFAB1, NDUFB10, NDUFB1, NDUFB2, NDUFB3, NDUFB4, NDUFB5, NDUFB6, NDUFB7, NDUFB8, NDUFB9, NDUFC1, NDUFC2, NDUFS4, NDUFS5, NDUFS6, NDUFV3, NDUFAF1, NDUFS1, NDUFS2, NDUFS3, NDUFS7, NDUFS8, NDUFV1, NDUFV2, SDHC, UQCR10, UQCRB, UQCRC1, UQCRC2, UQCRH, COX10, COX15, COX4I1, COX4I2, COX5A, COX8A, COX8C, ATP5C1, ATP5D, ATP7A, C1orf177 (LEXM), CHCHD10, COQ7, COQ9, CYCS, DLD, DNAJC15, FXN, GADD45GIP1, GBAS, MECP2, MLXIPL, MSH2, MYOG, PARK7, PINK1, PMPCB, PPIF, SDHAF2, SLC25A23, SLC25A33, SNCA, SURF1, TAZ, UQCRHL, VCP, ACTN3, AK2 and APOC3*), the gene list taken from (Frederick et al., 2020), and stress response/neuroprotection (*FKBP5, CRH, HSPA2, NR3C1, NR3C2, PPARA, PPARG, PPARD, RXRA, NPY and GPR37*), the gene list taken from (Mamdani et al., 2015), were extracted from the GTEx portal: https://gtex.portal.org/home/multiGeneQueryPage (Lonsdale et al., 2013). Gene expression levels are based on sample sizes between min. *n* = 114 (SN) to max. *n* = 209 (CRBL; GTEx portal).

Age‐related changes in gene expression were extracted from exon microarray data generated by the UKBEC (data set identifier GEO GSE46706). Sample demographics, procedures, DNA microarray analysis and quality control measures were described before (Trabzuni et al., 2011).

### 4.5 Generation of cell‐type dataset for expression‐weighted cell‐type enrichment analysis

The reference cell‐type dataset (https://github.com/RHReynolds/MarkerGenes) was generated using data from the Allen Institute for Brain Science (AIBS) study of 49,495 single nucleus transcriptomes across multiple human cortical areas (https://portal.brain‐map.org/atlases‐and‐data/rnaseq). All sampled brain regions were included and nuclei were annotated to cell types at two levels, with increasing resolution. Level 1 cell types included 7 types of glutamatergic neuron, 5 types of GABAergic neuron and 5 non‐neuronal cell types. Nuclei labelled as endothelial cells (*n* = 70), pericytes (*n* = 32) and VLMC (*n* = 11) were merged into the level 1 cell type, “vascular cell.” Level 2 cell types represented the 120 clusters originally identified in the dataset. Nuclei that were labelled as “outlier calls” in the accompanying metadata (*n* = 1985) were excluded. In addition, the following filtering steps were applied to genes: (i) removal of any genes symbols from the gene‐cell matrix that were not official HGNC symbols using EWCE::fix_bad_hgnc_symbols() (*n* = 30,792 genes were retained); (ii) removal of genes not expressed across any cell types (*n* = 1263); and (iii) removal of genes that were not significantly differentially expressed across level 2 cell types (*n* = 6304). Steps (ii) and (iii) were achieved using EWCE::drop_uninformative_genes(). The default LIMMA method was used together with an adjusted *p*‐value threshold for differential expression of *p* < 1e‐5. The filtered matrix was then run through EWCE::generate_celltype_data() to generate specificity values. Using this data, the proportion of total expression of a gene found in one cell type compared with all cell types was generated using the “generate.celltype.data” function of the EWCE package.

### 4.6 Expression‐weighted cell‐type enrichment analysis of pathways of interest

Expression‐weighted cell‐type enrichment (EWCE v. 1.4.0) (Skene & Grant, 2016) was used to determine whether genes associated with pathways/cellular processes of interest had significantly higher expression in certain cell types than might be expected by chance. The input target gene list, that is, all genes associated with relevant pathways listed in “Gene Expression Analysis” section were used as input. Gene names in the list were adjusted to match HGNC nomenclature using the R library HGNChelper (v. 0.8.1). Bootstrap gene lists with comparable properties to the target list (transcript length and GC‐content) were generated with EWCE iteratively (*n* = 10,000) using bootstrap_enrichment_test function. In brief, this function takes the inquiry gene list and a single cell‐type transcriptome data set and determines the probability of enrichment of this list in a given cell type when compared to the gene expression of bootstrapped gene lists; the probability of enrichment and fold‐change of enrichment are the returned. *P*‐values were corrected for multiple testing using the Benjamini–Hochberg method. Finally, enrichment plots were generated using the ewce_plot function. Values inferior to 0 are displayed as 0. This analysis was run in R 4.2.0.

## AUTHOR CONTRIBUTIONS

SRS, DAK, NWW, KB and KT conceived this project. NWW, KB and MR supplied tissue samples. LS supplied the UKBEC dataset. SRS performed sample preparation and MM‐qPCR, and SRS and KT analysed PCR dataset. SRS analysed GTEx data and generated figures. TG and KT performed TRF analysis. MGP and MR performed EWCE analysis. KT analysed UKBEC data and generated figures. SRS and KT developed the project and drafted the manuscript, and all authors contributed to the correction and approved.

## CONFLICT OF INTEREST STATEMENT

KPB received speaker honoraria from Ipsen, Merz, MDS; personal compensation for scientific advisory board for Mitsubishi (Neuroderm), Jazz Pharma and Ipsen; receives royalties from the publication of Oxford Specialist Handbook of Parkinson's Disease and Other Movement Disorders (Oxford University Press, 2008), Cambridge Press; and editorial work stipend from MDS for MDCP journal. SRS received personal compensation for scientific advisory board for Elemind Inc. All other authors report no competing interests.

## Supporting information


Appendix S1:

## Data Availability

The raw data that support the findings of this study are available from the corresponding authors upon reasonable request. Demographic and clinical details of human control spleen‐brain tissue donors used in this study are available from Table S1. Gene expression data used in this study are available from GTEx portal and from (Trabzuni et al., 2011).
