# Supplementary material for: Age‐related telomere attrition in the human putamen
Source: Aging Cell. 2023 May 2;22(7):e13861. doi: 10.1111/acel.13861 (PMC10352551; doi:10.1111/acel.13861)
Supplement: Supplementary file 1 — Appendix S1: [file ACEL-22-e13861-s001.pdf]

Age-related telomere attrition in the human putamen

Sebastian R. Schreglmann [1,2 *], Tomas Goncalves [3,4], Melissa Grant-Peters [5], Demis A. Kia [1], Lilach Soreq [1], Mina Ryten [5,6], Nicholas W. Wood [1], Kailash P. Bhatia [1], Kazunori Tomita [3,4 *]

1 Queen Square Institute of Neurology, University College London, UK

2 Department of Neurology, University Hospital Würzburg, Würzburg, Germany

3 Chromosome Maintenance Group, UCL Cancer Institute, University College London, UK

4 Centre for Genome Engineering and Maintenance, College of Health, Medicine and Life Sciences, Brunel University London, UK

5 Genetics and Genomic Medicine, Great Ormond Street Institute of Child Health, University College London, London

6 NIHR Great Ormond Street Hospital Biomedical Research Centre, University College London, London, UK

[*]Corresponding Authors

Sebastian R Schreglmann: sebastian.schreglmann@gmail.com

Kazunori Tomita: Kazunori.Tomita@brunel.ac.uk

Figure S1: Correlation between Mean T/S and TRFs

Figure S2: Region specific telomere length

Figure S3: Cell-type specific gene expression analysis

Figure S4: Correlation between regional telomere length and *TERF2* expression

Figure S5: Correlation between age or telomere length and *TERF2* expression

Table S1: Donor information and telomere length for putamen and spleen samples

Table S2: Information for “supratentorial” samples

Table S3: Age-related changed in expression level of genes involved in telomere maintenance and stress responses

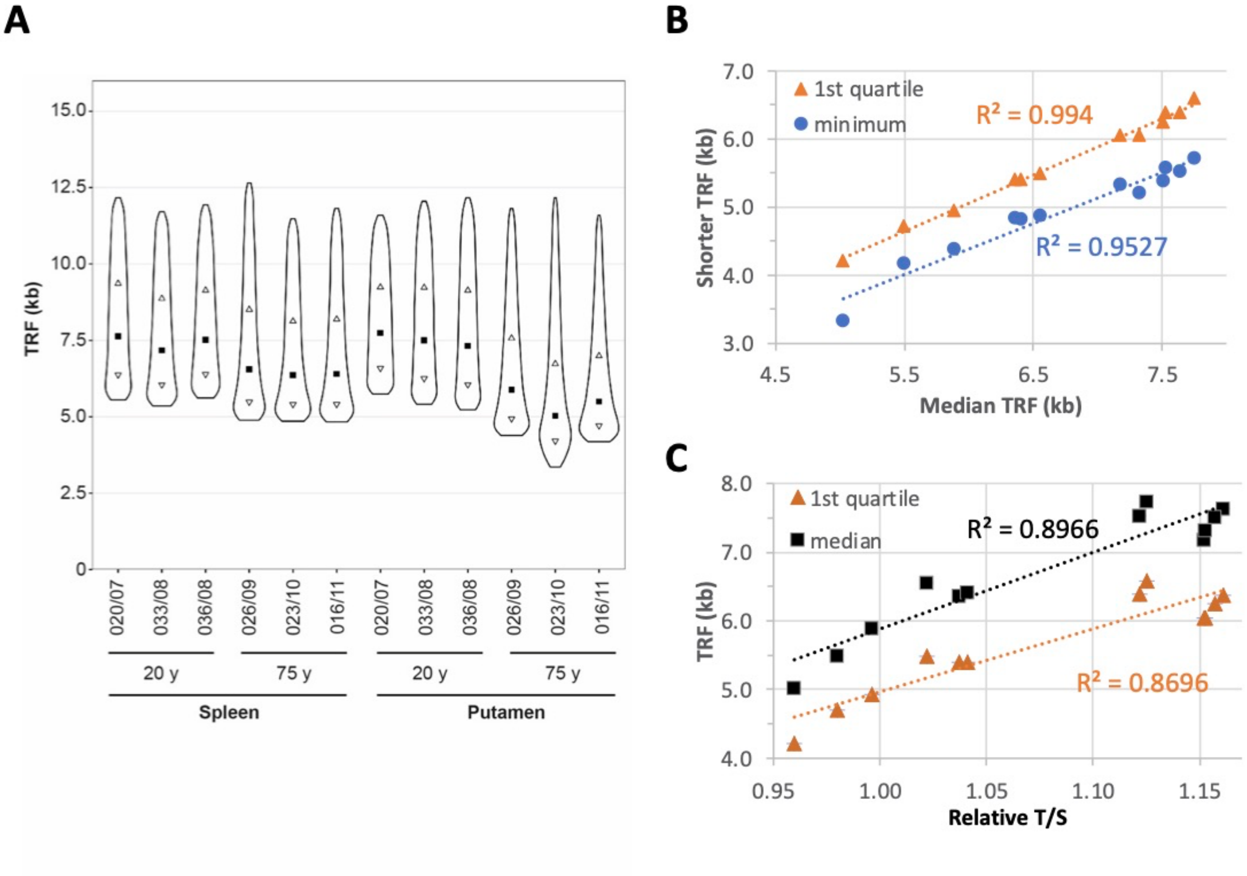

Figure S1: Correlation between Mean T/S and TRFs

(A) A violin graph includes minimal, 1st quartile (inverted triangle), median (closed square), 3rd quartile (triangle) and maximum lengths of TRFs from the blot in Figure 1A.

(B) A scatter plot shows that minimal (blue) and 1st quartile (orange) telomere length are proportional to its median (minimal: R^2=0.953, p=5.94x10^{-8}; 1st quartile: R^2=0.994, p=1.95x10^{-12}).

(C) A scatter plot shows the correlation between relative T/S and TRFs of median (black) and 1st quartile (orange) TRFs (median: R^2=0.897, p=3.05x10^{-6}; 1st quartile: R^2=0.870, p=9.85x10^{-6}).

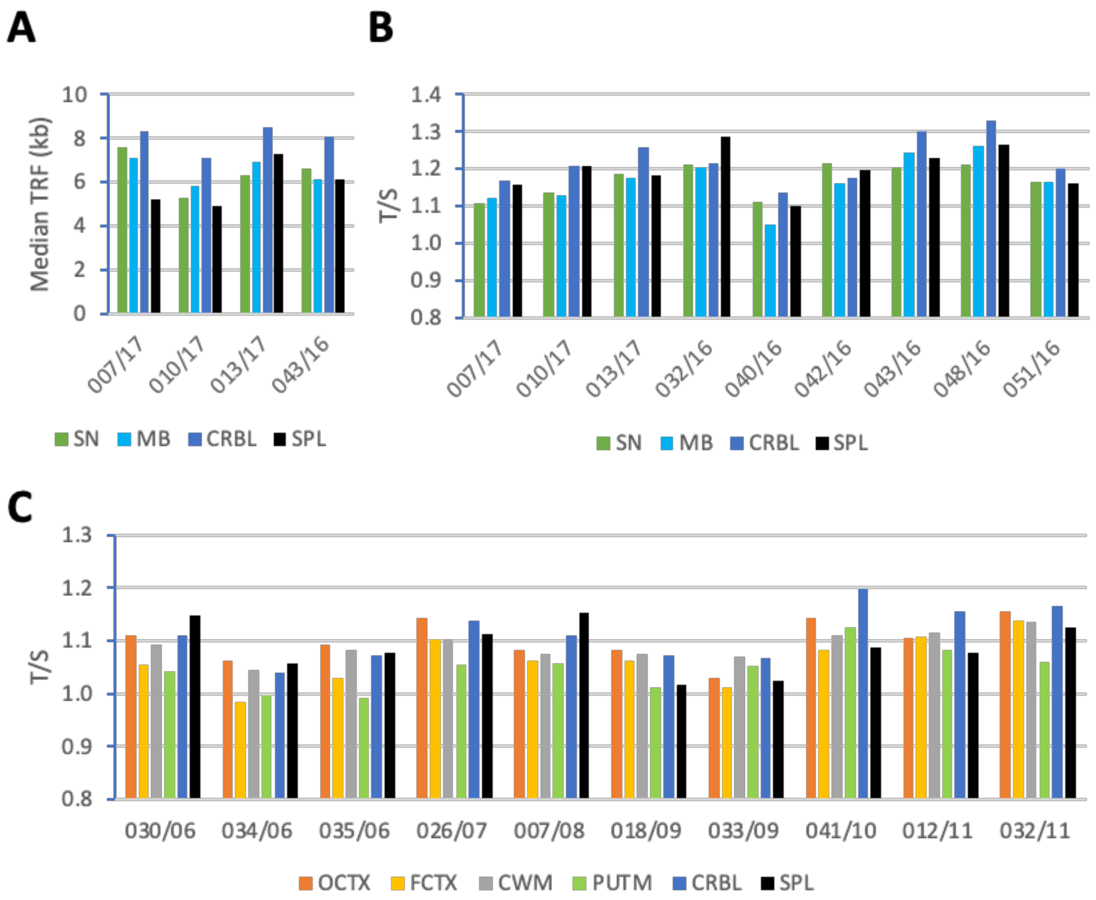

Figure S2: Region specific telomere length

A. A bar graph shows the median telomere length from the TRF analysis in Figure 2A.

(B, C) Bar graphs show relative mean telomere length (T/S) assessed by MM-qPCR.

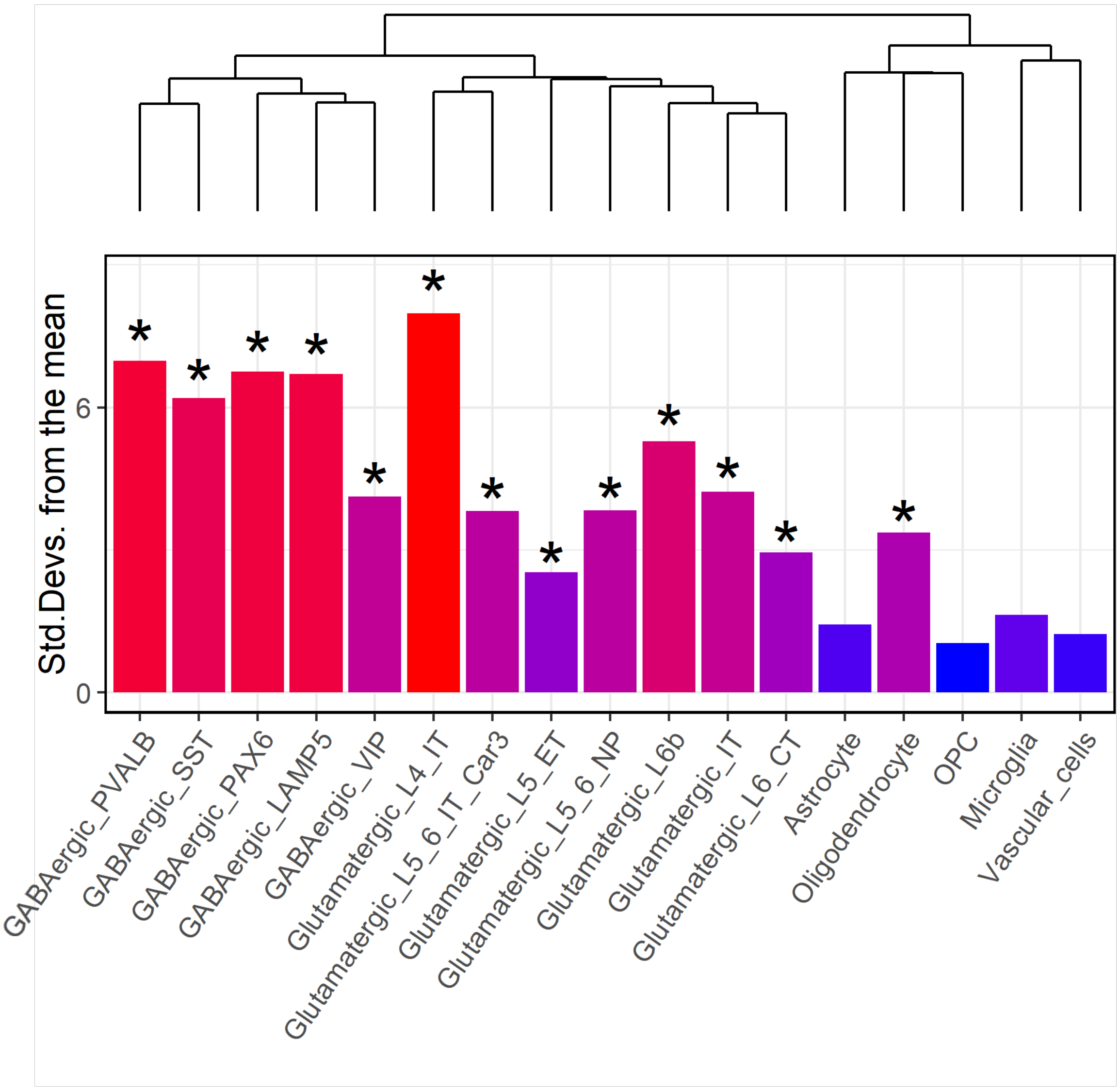

Figure S3: Cell-type specific gene expression analysis

Using a reference cell type dataset built on 49,495 single nucleus transcriptomes, expression-weighted cell-type enrichment analysis (EWCE) was performed. The graph shows significant enrichment for telomere length inheritance/maintenance/biogenesis, oxidative phosphorylation and stress response gene categories in neurons and oligodendrocytes, but not other glial cell types. Column colour shows the level of enrichment (low = blue, high = red). The asterisk indicates significant enrichment.

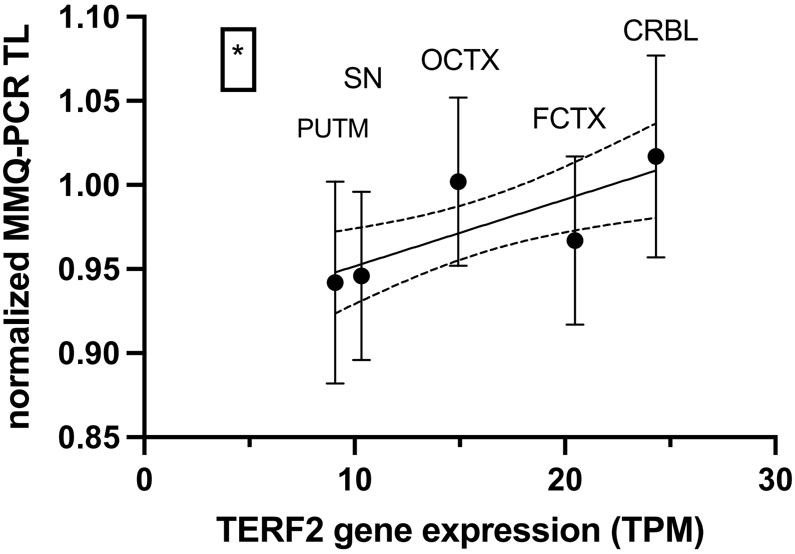

Figure S4: Correlation between regional telomere length and *TERF2* expression

Across studied brain regions, TERF2 expression based on GTEx RNA expression data from n=114 (SN) to n=209 (CRBL) samples correlated with the mean normalised telomere length in n=9 samples per region (simple linear regression, R^2=0.152, F=8.601, p=0.0051). * indicates p<0.01.

Table S1: Donor information and telomere length for putamen and spleen samples

Demographic and clinical details of human control spleen-brain tissue donors arranged by age at death as collected by the MRC Sudden Death Brain and tissue bank, Edinburgh. Cause of death has been ranked by the tissue donor bank by most immediate cause of death in numerical-alphabetical order. Mean T/S-values per sample are given for spleen (SPL) and putamen (PUTM) and rounded to three digits. Tissue collections "supratentorial" comprise of samples from donors 030/06, 034/06, 035/06, 026/07, 007/08, 018/09, 033/09, 041/10, 012/11 and 032/11. Abbreviations: post-mortem interval (PMI), hours (hrs).

Donor	Sex	Age	pH	PMI (hrs)	Cause of Death	Neuropathology report	Known medical records	SPL (T/S)	PUTM (T/S)
036/08	F	20	6.5	40	1a - Suspension by ligature	No significant abnormality	Depression, Alcohol abuse, Amlodipine 5mg daily, Bendrofluazide 2.5mg daily, Aspirin EC 300mg daily, Perindopril 2mg daily, Simvastatin 40mg nocte	1.122	1.153
020/07	F	22	6.36	44	1a - Butane inhalation	No significant abnormality	Alcohol & solvent abuse, Drug misuse, Depression, smoker	1.161	1.125
033/08	M	22	6.6	95	1a - Bilateral pulmonary embolism, 1b - Deep vein thrombosis, 2 - Combined effects of hypertensive heart disease and ischaemic heart disease, 2 - Obesity	No significant abnormality	Smoker, Asthma On no prescribed medication at time of death,	1.152	1.157
023/08	F	24	6.4	47	1a - Suspension by ligature	No significant abnormalities	Depression - recurrent	1.226	1.166
006/09	M	25	6.4	81	1a - Multiple Injuries, 1b - Road traffic collision (passenger)	No significant abnormalities	On no prescribed medication at time of death	1.140	1.120
014/09	M	26	6.3	44	1a - Methadone and diazepam toxicity	No significant abnormality	No medical history	1.099	1.104
021/10	M	27	6.2	67	1a – MDMA (ecstasy) toxicity, 2 - Ischaemic heart disease, coronary artery atherosclerosis and Type 1 Diabetes	No significant abnormality	Type 1 Diabetes	1.111	1.071
025/05	M	28	6.6	38	1a - Suspension by ligature	No significant abnormalities	Occasionally used cannabis	1.108	1.143
030/11	M	30	6.4	71	1a - Unascertained	No significant abnormalities	On no prescribed medication at time of death	1.178	1.086
035/09	M	32	6.2	99	1a - Suspension by ligature	No significant abnormalities	Chlorpromazine 50mg, Fluoxetine 20mg daily, Venlafaxine 150mg daily, Trazodone 200-300mg daily	1.254	1.157
006/10	F	32	6.3	63	1a - Acute decompensation of mitral valve disease and cardiomyopathy, 1b - Marfan's Syndrome	No significant abnormalities	Marfan Syndrome, Ramipril at time of death, Bisoprolol at time of death	1.141	1.127

032/10	M	34	6.4	57	1a - Sudden adult cardiac death	Old microscopic cerebellar infarct	On no prescribed medication at time of death	1.144	1.026
020/10	M	36	6.4	41	1a - Cardiomegaly,<br>1b - Ischaemic heart disease	No significant abnormalities	Smoker,<br>On no prescribed medication at time of death	1.039	1.119
025/08	M	37	6.4	63	1a - Ischaemic heart disease,<br>1b - Coronary artery atherosclerosis	Hyaline arteriosclerosis	On no prescribed medication at time of death	1.155	1.142
002/10	M	38	6.3	49	1a - Right ventricular hypertrophy	No significant abnormalities	On no prescribed medication at time of death	1.049	1.035
010/10	M	39	6.3	81	1a - Sudden Adult cardiac Death	No significant abnormalities	Depression - Venlafaxine	1.098	1.094
012/10	M	40	6.2	48	1a - Pulmonary thromboembolism<br>1b - Deep vein thrombosis<br>2 - Rupture of Achilles tendon	No significant abnormalities	No medical records as untraceable	1.052	1.052
007/06	M	41	6.4	49	1a - Dilated cardiomyopathy	No significant abnormalities	On no prescribed medication at time of death	1.101	1.137
006/08	M	41	6.26	50	1a - Ischaemic heart disease<br>1b - Coronary artery thrombosis<br>1c - Coronary artery atheroma<br>2 - Hypertensive heart disease	No significant abnormalities	On no prescribed medication at time of death	1.063	1.091
041/11	M	42	6.4	61	1a - Ischaemic heart disease,<br>1b - Coronary artery atherosclerosis	No significant abnormalities	Depression.<br>Prescribed medication at time of death: Dihydrocodeine 120mg tds, Terbinafine 250mg, Gabapentin 900mg bd	1.062	1.069
028/10	M	43	6.1	44	1a - Ischaemic heart disease,<br>1b - Coronary artery atherosclerosis	No significant abnormalities	On no prescribed medication at time of death	1.068	1.081
040/10	M	43	6.3	53	1a - Sudden cardiac death	No significant abnormalities	On no prescribed medication at time of death	1.109	1.082
029/11	F	43	6.00	86	1a - Bronchial asthma,<br>2 - Pulmonary congestion	No significant abnormalities	Asthma, Depression, Hypertension,<br>Medication prescribed at time of death: Amlodipine 5mg, Enalapril 10mg, Symbicort inhaler, Salbutamol inhaler	1.074	1.018
021/08	M	44	6.3	83	1a - Suspension by ligature	No significant abnormalities	Amitriptyline, Diazepam for stress	1.053	1.067
036/09	F	44	6.3	49	1a - Combined methadone, temazepam and codeine toxicity	No significant abnormalities	Depression, migraine<br>Propranolol 40mg tds, Paroxetine 30mg daily	1.109	1.079
018/05	M	45	6.4	75	1a - Ischaemic heart disease,<br>1b - Coronary artery atheroma,<br>1c - Atherosclerosis,<br>2 - Chronic renal failure	No significant abnormalities	On no medication at time of death.	1.053	1.037
009/07	M	45	6.2	67	1a - Myocardial infarction,<br>1b - Coronary artery thrombosis,<br>1c - Coronary artery atheroma	No significant abnormalities	Epigastric reflux, lansoprazole 15mg	1.142	1.058
002/08	F	45	6.4	28	1a - Ischaemic heart disease,<br>1b - Severe coronary artery atheroma	No significant abnormalities	Non-smoker,<br>Citalopram 10mg at time of death for depression	1.113	1.090
004/08	M	45	6.46	43	1a - Combined effects of hypertensive heart disease and ischaemic heart disease,<br>2-Diabetes (Type 1)	Early ischaemic neuronal injury	Hypertension, Type 1 Diabetes, Simvastatin, Lantus insulin, Loratadine, Lisinopril	1.047	1.011

026/08	M	45	6.4	39	1a - Ischaemic heart disease, 1b - Coronary artery thrombosis and atherosclerosis	No significant abnormalities	On no prescribed medication at time of death	1.197	1.084
038/08	M	46	5.9	91	1a - Ischaemic heart disease, 1b - Coronary artery atherosclerosis, 2 - Diabetes Mellitus	Small vessel disease, No other significant abnormality	Type 1 Diabetes, Atorvastatin 10mg for Hypercholesterolaemia	1.063	1.029
019/10	M	47	6	93	1a - Acute Myocardial Reinfarction, 1b - Previous Small Infarctions, 1c - Focally Advanced Atherosclerosis	Atherosclerosis small vessel disease	On no prescribed medication at time of death	1.117	1.055
040/08	M	48	6.5	59	1a - Ischaemic heart disease, 1b - Coronary artery atherosclerosis, 2 - Hypertensive heart disease	Small vessel disease in the form of hyaline arteriosclerosis, No other significant abnormality	Hypercholesterolaemia, Hypertension, Ramipril 5mg at time of death prescribed for Hypertension	1.066	0.998
024/09	M	48	5.4	67	1a - Myocardial infarction,1b - Coronary artery atherosclerosis (Angioplasty 12/05/09)	Small vessel disease, Cerebellar ischaemia	Smoker. Pizotifen 3mg at time of death for migraine, Omeprazole 20mg at time of death for Dyspepsia	1.125	1.056
039/10	M	48	6.2	95	1a - Myocardial re-infarction, 1b - Previous myocardial infarcts, 1c - Coronary atherosclerosis, 2 - Hypertensive heart disease	No significant abnormality	Smoker, On no prescribed medication at time of death, 28-30 units of alcohol weekly	1.169	1.146
036/11	M	48	6.4	72	1a - Coronary artery atherosclerosis	No significant abnormalities	Depression, On no prescribed medication at time of death.	1.021	1.025
037/11	M	48	6.2	46	1a - Coronary artery atherosclerosis	No significant abnormalities	diagnosed with Crohn's Disease, Prescribed Mesalamine 400mg qds for Crohn's at time of death	1.163	1.134
034/09	M	49	6.23	79	1a - Ischaemic and hypertensive heart disease	Neuropathological examination has demonstrated small vessel disease with evidence of microhaemorrhages, No significant acute pathology is identified	Asthma, On no prescribed medication at time of death	1.147	1.131
003/08	M	50	6.59	44	1a - Myocardial infarction, 1b - Severe coronary artery disease	No significant abnormalities	Smoker, On no prescribed medication at time of death	1.141	1.010
028/09	M	50	6.5	46	1a - Ischaemic heart disease, 1b - Coronary artery atherosclerosis	No significant abnormalities	Smoker, On no prescribed medication at time of death.	1.157	1.119
004/10	M	50	6.2	63	1a - Ischaemic heart disease	No significant abnormalities	Hypertension, Ischaemic heart disease, Atenolol 100mg, GTN spray, Amlodipine 10mg, Atorvastatin 40mg, Aspirin 75mg, Isosorbide Mononitrate 20mg	1.044	1.083
018/11	F	50	6.2	51	1a - Peritonitis, 1b - Bowel infarction	Small vessel disease No other significant abnormality	Smoker, On no prescribed medication at time of death.	1.134	1.000
017/08	F	51	6.3	41	1a - Unascertained	No significant abnormalities	On no prescribed medication at time of death	1.150	1.106

020/08	M	51	6.5	47	1a - Ischaemic heart disease,<br>1b - Coronary arteriosclerosis	Small vessel disease<br>No significant abnormality	Non-smoker,<br>On no prescribed medication at time of death	1.088	1.022
031/09	M	51	6.3	84	1a - Ischaemic heart disease,<br>1b - Coronary artery atherosclerosis	Small vessel disease	Hypercholesterolaemia,<br>Bezafibrate 400mg nocte,<br>Simvastatin 80mg daily	1.141	1.083
033/10	M	51	6.3	52	1a - Ischaemic and hypertensive heart disease	No significant abnormalities	Hypertension,<br>Carbimazole 15mg tds,<br>Felodipine 5mg daily	1.080	1.029
004/06	F	52	5.7	63	1a - Acute myocardial infarct,<br>2 - Dilated cardiomyopathy	No significant abnormalities	On no prescribed medication at time of death	1.172	1.187
015/10	M	52	6.2	47	1a - Coronary thrombosis,<br>1b - Ischaemic heart disease,<br>1c - Advanced coronary atherosclerosis	No significant abnormalities	On no prescribed medication at time of death	1.059	1.007
036/10	M	52	6.4	52	1a - Ischaemic and hypertensive heart disease	No significant abnormalities	Hypertension,<br>Hypercholesterolaemia,<br>Alcohol abuse,<br>Lisinopril 10mg daily,<br>Simvastatin 20mg daily	1.252	1.099
022/06	M	53	6.3	51	1a - Acute myocardial infarct,<br>1b - Severe coronary artery atheroma<br>2 - Dilated cardiomyopathy<br>2 - Hypertensive heart disease	Hypoxic brain damage,<br>Global cerebral ischaemia,<br>Mineralisation of hippocampal and basal ganglia vessels	Moderate smoker and drinker who took regular exercise,<br>On no medication at time of death	1.100	0.983
030/06	M	53	6.4	43	1a - Combined effects of ischaemic heart disease and hypertensive heart disease	Small vessel disease consistent with chronic hypertension.<br>No other significant abnormalities	Hypercholesterolaemia,<br>On no prescribed medication at time of death.	1.146	1.039
034/06	M	53	6.4	36	1a - Suspension by ligature	No significant abnormalities	Hypercholesterolaemia,<br>Hypertension,<br>Atorvastatin 80mg at time of death, Atenolol 25mg at time of death, Aspirin 75mg at time of death	1.085	1.024
043/06	F	53	6.38	42	1a - Ischaemic heart disease,<br>1b - Coronary artery atheroma,<br>1c - Atherosclerosis,<br>2 - Chronic obstructive pulmonary disease	No significant abnormalities	On no prescribed medication at time of death.	1.075	1.084
012/11	M	53	6.3	42	1a - Ischaemic heart disease,<br>1a - Coronary artery atherosclerosis	No significant abnormalities	On no prescribed medication at time of death. Untraceable on NHS system, therefore no medical records.	1.054	1.003
035/06	M	54	6.3	69	1a -Ischaemic heart disease,<br>1b- Severe coronary artery atheroma,<br>2- Chronic obstructive pulmonary disease	No significant abnormalities	No medication at time of death.	1.075	0.988
007/08	M	55	6.5	51	1a - Combined effects of hypertensive heart disease and ischaemic heart disease<br>2 - Chronic obstructive pulmonary disease	No significant abnormalities	On no prescribed medication at time of death	1.151	1.055
033/09	M	56	6.2	61	1a - Acute myocardial infarction,<br>1b - Coronary thrombosis,<br>1c - Coronary artery disease<br>2 - Hodgkin's disease on therapy	No significant abnormalities	Hypercholesterolaemia,<br>Simvastatin 40mg daily,<br>Omeprazole,<br>Dihydrocodeine	0.967	1.004

026/07	M	57	6.4	53	1a - Ischaemic heart disease, 1b - Coronary artery atheroma, 1c - Atherosclerosis, 2 - Hypertensive heart disease	Lipohyalinosis (chronic hypertension)	On no prescribed medication at time of death	1.109	1.052
022/08	F	57	6.5	95	1a - Pulmonary thromboembolism	Small vessel disease	Depression, Alcohol abuse, Venlafaxine 75mg daily, Antabuse	1.142	1.077
018/09	M	57	6.2	49	1a - Ruptured abdominal aortic aneurysm	No significant abnormalities	Non-smoker, Simvastatin 40ng at time of death, Candesartan 8mg at time of death	1.013	0.969
041/10	M	57	6.5	66	1a - Ischaemic heart disease, 1b - Coronary artery atherosclerosis	No significant abnormalities	On no prescribed medication at time of death	1.085	1.044
019/08	M	58	5.5	69	1a - Myocardial infarction,1b - Small previous infarctions,1c - Coronary arteriosclerosis,2 - General arteriosclerosis	Hyaline arteriosclerosis (small vessel disease), No significant abnormality	No GP Notes available (possibly destroyed).	1.154	1.057
013/11	M	58	6.2	137	1a - Ischaemic heart disease, 1b - Coronary artery atherosclerosis, 2 - Pulmonary congestion and oedema	No significant abnormalities	Smoker, Hypertension Bendrofluazide, Atenolol and Simvastatin at time of death.	1.137	1.118
032/11	M	58	6.4	43	1a - Acute myocardial infarction, 1b - Coronary thrombosis, 1c - Coronary artery atherosclerosis	Cerebrovascular disease	On no prescribed medication at time of death.	1.123	1.058
025/09	M	59	6.3	51	1a - Cardiac tamponade, 1b - Myocardial re-infarction, 1c - Coronary artery thrombosis, 1d - Ischaemic heart disease, 2 - Chronic hypertension	No significant abnormalities	Smoker, Asthma, Hypertension, Lansoprazole daily at time of death	1.012	1.040
015/07	M	60	6.54	66	1a - Ischaemic heart disease, 1b - Coronary artery atheroma	No significant abnormalities	Smoker, Hypertension, Alcohol abuse, Ramipril at time of death	1.058	1.045
022/09	F	60	6.3	36	1a - Hypertensive heart disease, 2 - Morbid obesity	Cerebrovascular disease, Small vessel disease, atherosclerosis, lacunar infarcts	Smoker, Temazepam, Doxepin, Salbutamol inhaler all at time of death	1.190	1.081
032/09	F	60	5.4	38	1a - Haemopericardium, 1b - Ruptured myocardial infarction, 1c - Coronary artery atherosclerosis, 2 - Hypertensive heart disease	No significant abnormalities	Smoker, Hypertension, Hypercholesterolaemia, Enalapril 20mg daily, Xipamide 20mg daily, Atenolol 100mg daily, Cerivastatin	1.129	1.038
038/09	M	60	6.4	41	1a - Ischaemic heart disease, 1b - Coronary artery atherosclerosis	Cerebrovascular disease, Atherosclerosis, Small vessel disease, Subacute infarction	EX smoker, On no prescribed medication at time of death	1.072	1.057
011/10	M	60	6.3	40	1a - Coronary artery thrombosis 1b - Ischaemic heart disease	Small vessel disease	EX smoker. Aspirin 75mg daily, Atenolol 25mg daily, GTN spray	1.016	1.042
013/10	M	60	6.3	68	1a - Coronary artery thrombosis, 1b - Coronary artery atherosclerosis, 2 - Hypertensive heart disease	No significant abnormalities	Hypercholesterolaemia, Hypertension, Simvastatin 20mg, Atenolol 100mg	1.089	1.080

018/08	M	61	6.5	43	1a - Ruptured coronary artery atherosclerotic plaque, 1b - Coronary artery atherosclerosis	No significant abnormalities	On no prescribed medication at time of death	1.202	1.085
030/09	F	61	6.1	70	1a - Coronary artery atherosclerosis (coronary angiography and angioplasty	No significant abnormalities	Hypercholesterolaemia, Hypertension, Ischaemic heart disease, Atenolol 50mg, Aspirin 75mg, Simvastatin 40mg, Isosorbide Mononitrate 20mg	1.084	1.098
008/10	M	61	6.2	47	1a - Ischaemic heart disease, 1b - Coronary artery atherosclerosis	Small vessel disease	On no prescribed medication at time of death	1.028	0.991
014/10	M	61	6.2	47	1a - Ischaemic heart disease, 1b - Coronary artery atherosclerosis	No significant abnormalities	Smoker ,Hypertension, Bendrofluazide 2.5mg at time of death	1.046	0.996
012/08	M	62	6.5	67	1a - Ischaemic heart disease, 1b - Previous myocardial infarctions,1c - Advanced coronary arteriosclerosis	No significant abnormalities	Hypercholesterolaemia, Simvastatin, Aspirin	1.137	1.125
001/10	M	62	6.1	35	1a - Haemopericardium, 1b - Ruptured myocardial infarction, 1c - Coronary artery thrombosis, 1d- Coronary artery atherosclerosis	Old infarct (Hypothalamus), No other significant abnormality	Not on any prescribed medication at time of death	1.076	1.093
014/11	M	62	6.3	135	1a - Ruptured abdominal aortic aneurysm, 2 - Hypertensive heart disease, 2 - Chronic obstructive pulmonary disease	Cerebrovascular disease, Atherosclerosis, Lipohyalinosis, Old microscopic frontal infarct	Asthma, hypertension, hypercholesterolaemia, stopped smoking 2006. Symbicort inhaler at time of death, Salbutamol inhaler at time of death, Diclofenac at time of death, Atorvastatin at time of death.	1.038	0.997
026/11	M	62	6.2	46	1a - Pontine haemorrhage, 1b - Hypertensive heart disease	Pontine hypertensive haemorrhage, Small vessel disease	Smoker. On no prescribed medication at time of death	1.076	1.006
016/10	F	63	6.3	35	1a - Ischaemic heart disease, 1b - Advanced Coronary Atherosclerosis	No significant abnormality	Diagnosed with coeliac disease, was smoking 10-20 cigarettes daily, drinking 6 units alcohol 28/02/2000: diagnosed with osteoporosis. 04/07/2008: Hypertensive at 194/111 and lipids high 02/04/2009: had been diagnosed with a liver lesion that was initially thought to be metastatic disease from an unknown primary. However, liver lesion was now confirmed as a haemangioma. Commenced on Amlodipine and a statin. Alendronate 10mg daily, Amlodipine ??, Simvastatin ??	0.995	1.050
011/11	M	63	6.3	67	1a - Hypertensive and ischaemic heart disease, 1b - Coronary artery atherosclerosis	Small vessel disease	Hypertension, Bendrofluazide 2.5mg at time of death, Coracten XI M/R 30 mg daily at time of death.	1.010	0.947

023/09	F	64	6.6	48	1a - Ischaemic heart disease,<br>1b - Coronary artery atherosclerosis,<br>2 - Hypertensive heart disease	No significant abnormalities	Hypertension.<br>Lisinopril 40mg at time of death, Bendrofluazide 2.5mg at time of death	1.131	1.033
021/09	M	65	6.4	40	1a - Ischaemic heart disease,<br>1b - Coronary artery atherosclerosis	No significant abnormalities	Smoker.<br>On no prescribed medication at time of death	1.061	1.009
003/10	M	65	5.6	34	1a - Diabetic Keto-acidosis and Morphine Toxicity,<br>2 - Ischaemic heart disease	Small vessel disease	Type 1 Diabetes, Hypertension, Smoker, Amitriptyline 150mg daily, Enalapril 5mg daily.	1.063	1.125
019/11	M	66	6.40	144	1a - Haemopericardium,<br>1b - Ruptured atherosclerotic aortic root aneurysm,<br>2 - Hypertensive heart disease	No significant abnormalities	1991 - osteoarthritis,<br>1993 - duodenal ulcer<br>Not on any prescribed medication.	1.048	1.047
020/11	M	66	6.3	81	1a - Cardiac tamponade,<br>1b - Ruptured myocardial infarct,<br>1c - Coronary artery atherosclerosis	Small vessel disease, Subacute lacunar infarct, Large vessel atherosclerosis	Non-smoker<br>On no prescribed medication at time of death.	1.074	1.022
025/11	M	66	6.4	69	1a - Cardiac tamponade,<br>1b - Ruptured myocardial infarct,<br>1c - Coronary artery atherosclerosis	No significant abnormalities	On no prescribed medication.	1.105	1.059
014/08	M	67	6.6	50	1a - Ruptured abdominal aortic aneurysm and coronary thrombosis,<br>1b - Atherosclerosis,<br>2 - Ischaemic heart disease,<br>2 - Chronic obstructive pulmonary disease	No significant abnormalities	Smoker<br>Lisinopril 2.5mg daily, Atorvastatin, Digoxin, Warfarin,	1.115	1.071
015/09	M	67	6.4	47	1a - Ischaemic heart disease,<br>1b - Coronary artery thrombosis,<br>1c - Coronary artery atherosclerosis	No significant abnormalities	Stopped smoking in 2005.<br>Minimal alcohol<br>On no prescribed medication at time of death	1.021	0.984
018/10	M	69	6.00	35	1a - Ischaemic & Hypertensive Heart Disease,<br>2 - Diabetes mellitus Type 1	Cerebrovascular disease	Type 2 Diabetes, Hypertension, Smoker, Metformin 850mg bd, Gliclazide 160mg am, 80mg pm, Adalat Retard 10mg bd	1.136	1,057
034/08	M	70	6.2	50	1a - Pulmonary embolism,<br>1b - Deep vein thrombosis,<br>2 - Previous pulmonary embolism,<br>2 - Hypertension	No significant abnormalities	Depression, Hypercholesterolaemia, Hypertension,<br>Lisinopril 5mg daily, Simvastatin 20mg daily	1,115	1,022
007/10	M	70	6.3	96	1a - Coronary thrombosis.<br>1b - Ischaemic heart disease.<br>2 - Chronic pulmonary emphysema	Small vessel disease, Lacunar infarcts	On no prescribed medication at time of death	1,025	1.008
033/11	F	72	6.3	30	1a - Haemopericardium,<br>1b - Ruptured myocardial infarct,<br>1c - Coronary artery thrombosis.<br>1d - Coronary artery atherosclerosis	No significant abnormalities<br>Vascular disease	No Medical History. Nil of relevance in GP notes	1,061	1.093
026/09	M	73	6.2	51	1a - Ischaemic heart disease,<br>1b - Coronary artery atherosclerosis	Small vessel disease, Old lacunar infarct.	Ischaemic heart disease, Smoker,<br>Simvastatin 10mg daily, Nifedipine 10mg daily, Aspirin 75mg daily, GTN spray	1.022	0.996

023/10	M	74	6.4	44	1a - Ischaemic heart disease, 1b - Coronary artery atherosclerosis	No significant abnormalities	On no prescribed medication at time of death	1,037	0.960
016/11	M	74	6.3	66	1a - Ischaemic heart disease, 1b - Coronary artery occlusion 1c - Coronary artery atherosclerosis,	Cerebrovascular disease, Large vessel atherosclerosis, Small vessel disease	Hypertension, Atenolol 100mg daily, Aspirin 75mg daily	1.041	0.980
025/10	M	75	5.4	47	1a - Ischaemic heart disease, 1b - Coronary atherosclerosis	Small vessel disease, No other significant abnormality	On no prescribed medication at time of death	1,090	1.030
013/09	F	79	6.3	45	1a - Ischaemic heart disease, 1b - Coronary artery atherosclerosis	Small vessel disease	Hypertension. Rheumatoid arthritis. Letter in notes detailing that she suffered from hypertension and hypothyroidism but no actual record of when she was diagnosed. Left ankle replacement. Thyroxine ?? Dosage, Tenoret 50 ??, Pulsatilla 6 2-3 daily prescribed ??	1.049	1.145

Table S2: Information for "supratentorial" samples

Demographic details of human control tissue donors for "supratentorial" samples, arranged by age at death as collected by the MRC Sudden Death Brain and tissue bank, Edinburgh. Abbreviations: post-mortem interval (PMI), hours (hrs).

Donor	Sex	Age	pH	PMI (hrs)
043/16	M	47	6,18	67
048/16	M	49	6,21	94
013/17	M	51	6,38	52
042/16	M	52	6,3	99
007/17	M	53	6,37	96
010/17	M	57	6,4	64
040/16	M	57	6,28	113
051/16	M	57	n.k.	110
032/16	M	58	6,14	104

Table S3: Age-related changed in expression level of genes involved in telomere maintenance and stress responses (the numeric information of Figure 4)

		Region	PUTM		SNIG		HIPP		CRBL		WHTM		FCTX		OCTX	
		N=	129		101		122		130		130		127		129	
Group	Gene name	Probe	*r*	p	*r*	p	*r*	p	*r*	p	*r*	p	*r*	p	*r*	p
1	PARP1	2458773	-0.27	-2.7	-0.21	-1.4	-0.22	-1.8	0.06	-0.3	0.10	-0.6	-0.12	-0.8	-0.09	-0.5
1	PRRC2A	2902463	-0.26	-2.5	-0.20	-1.3	-0.32	-3.6	-0.06	-0.3	0.00	0.0	-0.26	-2.5	-0.37	-4.8
1	CARMIL1	2898746	-0.25	-2.4	-0.19	-1.2	-0.02	-0.1	0.17	-1.2	-0.03	-0.1	0.25	-2.3	0.32	-3.7
1	ZBTB46	3914114	-0.23	-2.0	-0.01	-0.1	-0.25	-2.3	0.22	-1.9	-0.21	-1.8	-0.17	-1.3	0.24	-2.2
1	MPP6	2993206	-0.21	-1.8	-0.36	-3.7	-0.28	-2.7	0.37	-4.8	-0.16	-1.2	-0.07	-0.4	0.07	-0.4
1	STMN3	3914050	-0.13	-0.8	-0.25	-1.9	-0.28	-2.8	0.16	-1.1	-0.22	-1.9	0.02	-0.1	-0.12	-0.7
1	MOB1B	2730673	-0.03	-0.2	-0.02	-0.1	-0.14	-0.9	0.26	-2.5	0.17	-1.2	0.02	-0.1	0.04	-0.2
1	SENP7	2686646	0.05	-0.2	0.02	-0.1	0.23	-1.9	-0.03	-0.1	0.12	-0.7	0.03	-0.1	-0.14	-1.0
1	RNF201	3699044	0.09	-0.5	0.18	-1.2	0.28	-2.7	0.00	0.0	0.30	-3.2	0.14	-0.9	-0.02	-0.1
1	ZNF208	3856646	0.10	-0.6	-0.01	-0.1	0.04	-0.2	0.36	-4.5	0.25	-2.4	0.00	0.0	0.24	-2.2
1	DCAF4	3543355	0.15	-1.0	-0.15	-0.9	-0.03	-0.1	0.22	-1.9	0.15	-1.0	0.09	-0.5	0.09	-0.5
2	TERC	2704733	-0.35	-4.4	-0.27	-2.2	-0.50	-8.5	0.03	-0.1	0.02	-0.1	-0.30	-3.2	0.16	-1.2
2	GAR1	2739242	-0.24	-2.2	-0.44	-5.4	-0.45	-6.8	-0.04	-0.2	-0.32	-3.8	-0.28	-2.8	0.03	-0.1
2	TERT	2845829	-0.19	-1.5	-0.33	-3.2	-0.37	-4.7	-0.01	0.0	-0.21	-1.7	-0.26	-2.5	0.02	-0.1
2	DKC1	3996667	-0.15	-1.0	-0.12	-0.6	-0.04	-0.2	0.26	-2.5	0.28	-2.8	0.13	-0.9	0.14	-1.0
2	PARN	3681377	-0.09	-0.5	-0.29	-2.4	-0.02	-0.1	-0.19	-1.5	-0.08	-0.4	-0.17	-1.3	-0.21	-1.7
2	NOP10	3617403	-0.09	-0.5	-0.34	-3.3	-0.25	-2.3	-0.05	-0.3	-0.29	-3.2	-0.25	-2.4	-0.32	-3.6
2	NHP2	2889456	0.02	-0.1	-0.06	-0.3	-0.19	-1.4	-0.17	-1.3	0.06	-0.3	0.08	-0.4	0.08	-0.5
2	TCAB1	3708959	0.04	-0.2	0.08	-0.4	-0.01	0.0	0.03	-0.1	-0.01	0.0	-0.09	-0.5	-0.02	-0.1
2	NAF1	2792069	0.11	-0.7	0.02	-0.1	0.09	-0.5	-0.09	-0.5	0.14	-1.0	-0.02	-0.1	-0.14	-1.0
2	TEP1	3555340	0.22	-1.9	0.31	-2.7	0.46	-6.9	-0.10	-0.6	0.09	-0.5	0.31	-3.4	0.16	-1.2
3	TERF2	3696571	-0.44	-6.8	-0.48	-6.5	-0.40	-5.3	-0.02	-0.1	-0.11	-0.7	-0.20	-1.6	-0.14	-1.0
3	TERF2IP	3669092	-0.34	-4.2	-0.31	-2.8	-0.34	-3.8	0.05	-0.2	0.01	-0.1	-0.11	-0.6	-0.16	-1.1
3	TZAP	2318364	-0.34	-4.1	-0.23	-1.7	-0.27	-2.6	-0.07	-0.4	-0.02	-0.1	-0.18	-1.4	-0.26	-2.5
3	TEN1	3735346	-0.27	-2.8	-0.39	-4.4	-0.40	-5.2	0.15	-1.1	-0.16	-1.2	-0.36	-4.6	0.01	0.0
3	ACD	3665645	-0.19	-1.5	-0.23	-1.7	-0.31	-3.3	0.10	-0.6	-0.28	-2.9	-0.12	-0.8	0.01	0.0
3	RTEL1	3893520	-0.12	-0.8	0.12	-0.6	-0.02	-0.1	0.09	-0.5	-0.08	-0.4	-0.04	-0.2	0.05	-0.2
3	TINF2	3558012	-0.11	-0.7	-0.09	-0.4	0.11	-0.7	-0.04	-0.2	0.25	-2.3	-0.04	-0.2	-0.21	-1.8
3	ATR	2698844	-0.08	-0.4	-0.14	-0.8	-0.08	-0.4	-0.09	-0.5	0.16	-1.2	-0.07	-0.3	-0.15	-1.0
3	CTC1	3744314	-0.06	-0.3	0.26	-2.1	0.00	0.0	0.15	-1.0	0.15	-1.0	0.21	-1.7	0.07	-0.4
3	STN1	3305013	0.00	0.0	0.34	-3.3	0.26	-2.5	-0.02	-0.1	0.19	-1.6	-0.18	-1.3	-0.30	-3.3
3	RIF1	2510485	0.00	0.0	0.00	0.0	0.06	-0.3	0.01	0.0	0.24	-2.2	-0.01	-0.1	-0.11	-0.6
3	ATM	3390067	0.02	-0.1	-0.11	-0.5	0.12	-0.7	0.18	-1.4	0.11	-0.7	-0.02	-0.1	-0.09	-0.5
3	POT1	3070908	0.19	-1.5	-0.01	0.0	0.24	-2.1	0.02	-0.1	0.19	-1.6	0.07	-0.4	-0.09	-0.5
3	TERF1	3103187	0.20	-1.6	0.34	-3.3	0.29	-2.9	-0.02	-0.1	0.39	-5.2	0.29	-3.1	0.10	-0.6

4	NDUFB7	3852823	-0.54	-10	-0.51	-7.3	-0.50	-8.2	0.18	-1.4	-0.32	-3.7	-0.40	-5.6	-0.13	-0.9
4	PFKL	3923632	-0.52	-9.4	-0.26	-2.1	-0.34	-3.8	0.17	-1.3	0.02	-0.1	-0.10	-0.6	0.06	-0.3
4	UQCRC1	2673509	-0.51	-9.0	-0.37	-3.9	-0.38	-4.9	-0.01	0.0	-0.10	-0.6	-0.31	-3.4	-0.22	-1.9
4	NDUFV1	3337196	-0.49	-8.4	-0.39	-4.2	-0.42	-5.8	0.02	-0.1	-0.07	-0.4	-0.31	-3.4	-0.25	-2.4
4	PINK1	2324097	-0.49	-8.3	-0.38	-4.1	-0.45	-6.7	0.14	-0.9	-0.27	-2.7	-0.33	-3.7	-0.12	-0.7
4	UQCRH	2334689	-0.48	-8.2	-0.39	-4.3	-0.50	-8.5	-0.14	-0.9	-0.35	-4.3	-0.32	-3.6	-0.23	-2.0
4	ALDOC	3750767	-0.47	-7.7	-0.32	-3.0	-0.26	-2.4	0.10	-0.6	-0.25	-2.4	-0.29	-3.0	-0.19	-1.5
4	NDUFS3	3329904	-0.46	-7.4	-0.51	-7.3	-0.53	-9.3	0.00	0.0	-0.17	-1.3	-0.29	-2.9	-0.24	-2.2
4	PFKP	3232349	-0.45	-7.2	-0.32	-3.0	-0.30	-3.0	0.02	-0.1	-0.14	-1.0	-0.18	-1.4	-0.18	-1.4
4	ATP5D	3815610	-0.44	-6.8	-0.55	-8.5	-0.42	-5.9	-0.04	-0.2	-0.36	-4.6	-0.43	-6.3	-0.38	-5.1
4	NDUFA7	3848885	-0.44	-6.8	-0.41	-4.7	-0.36	-4.3	0.23	-2.1	0.04	-0.2	-0.29	-3.0	-0.24	-2.3
4	ENO1	2395490	-0.44	-6.8	-0.18	-1.1	0.09	-0.5	0.40	-5.5	0.07	-0.4	-0.04	-0.2	0.10	-0.6
4	COQ7	3650802	-0.44	-6.7	-0.24	-1.9	-0.39	-5.1	-0.14	-1.0	0.05	-0.2	-0.29	-3.0	-0.28	-2.8
4	NDUFB2	3027538	-0.43	-6.5	-0.51	-7.3	-0.48	-7.7	-0.01	0.0	-0.29	-3.0	-0.16	-1.1	-0.25	-2.3
4	GAPDH	3402625	-0.43	-6.3	-0.39	-4.3	-0.17	-1.2	-0.26	-2.5	-0.08	-0.5	-0.51	-9.0	-0.39	-5.3
4	HK1	3250278	-0.41	-6.0	-0.26	-2.1	-0.42	-5.8	0.00	0.0	0.03	-0.1	-0.21	-1.8	-0.10	-0.6
4	NDUFA8	3224197	-0.39	-5.4	-0.47	-6.2	-0.44	-6.5	0.12	-0.8	0.05	-0.2	-0.22	-1.9	-0.13	-0.8
4	CYCS	3042001	-0.39	-5.3	-0.45	-5.5	-0.50	-8.3	-0.21	-1.8	-0.26	-2.5	-0.28	-2.8	-0.12	-0.7
4	PFKFB3	3233605	-0.37	-4.7	-0.21	-1.4	0.19	-1.4	0.24	-2.3	0.05	-0.2	-0.11	-0.7	-0.05	-0.2
4	NDUFA1	3988987	-0.36	-4.6	-0.49	-6.8	-0.38	-4.9	0.01	-0.1	0.00	0.0	-0.11	-0.7	-0.08	-0.4
4	PGAM1	3259817	-0.36	-4.5	-0.40	-4.5	-0.43	-6.1	-0.18	-1.4	0.02	-0.1	-0.15	-1.0	-0.15	-1.1
4	NDUFAF1	3619991	-0.35	-4.3	-0.34	-3.3	-0.27	-2.6	-0.05	-0.2	0.06	-0.3	-0.21	-1.7	-0.20	-1.7
4	CHCHD10	3954887	-0.35	-4.3	-0.40	-4.5	-0.45	-6.7	0.15	-1.0	-0.15	-1.0	-0.15	-1.0	-0.13	-0.9
4	NDUFB8	3303530	-0.35	-4.3	-0.43	-5.2	-0.31	-3.3	0.07	-0.4	-0.24	-2.3	-0.09	-0.5	-0.02	-0.1
4	ALDOA	3655920	-0.34	-4.0	-0.30	-2.7	-0.29	-2.9	0.17	-1.3	-0.27	-2.7	-0.30	-3.2	-0.17	-1.3
4	NDUFA6	3962260	-0.33	-3.8	-0.41	-4.8	-0.34	-3.8	0.13	-0.8	-0.01	0.0	-0.21	-1.7	-0.08	-0.5
4	NDUFB1	3576937	-0.32	-3.7	-0.29	-2.4	-0.42	-5.8	-0.16	-1.2	-0.08	-0.4	-0.28	-2.9	-0.24	-2.1
4	PARK7	2318736	-0.31	-3.4	-0.24	-1.8	-0.30	-3.0	0.12	-0.8	0.01	0.0	0.03	-0.1	0.03	-0.1
4	NDUFS6	2799184	-0.31	-3.4	-0.41	-4.7	-0.35	-4.1	0.13	-0.9	-0.04	-0.2	-0.13	-0.8	0.17	-1.3
4	VCP	3204404	-0.30	-3.4	-0.26	-2.1	-0.25	-2.3	0.14	-1.0	0.13	-0.9	-0.03	-0.2	0.02	-0.1
4	NDUFA4	3038617	-0.30	-3.2	-0.54	-8.3	-0.39	-5.0	-0.16	-1.2	-0.20	-1.6	-0.35	-4.2	-0.31	-3.4
4	C1orf177	2337327	-0.30	-3.2	-0.12	-0.6	-0.22	-1.8	0.04	-0.2	-0.13	-0.8	-0.14	-0.9	0.16	-1.2
4	GADD45GIP1	3851911	-0.30	-3.2	-0.38	-4.0	-0.50	-8.5	0.17	-1.3	-0.20	-1.6	-0.20	-1.6	0.01	0.0
4	NDUFA5	3070658	-0.29	-3.1	-0.35	-3.4	-0.33	-3.8	-0.07	-0.4	-0.06	-0.3	-0.21	-1.7	-0.23	-2.0
4	PGK1	3982462	-0.29	-3.1	-0.32	-2.9	-0.34	-3.9	-0.10	-0.6	0.06	-0.3	-0.16	-1.2	-0.24	-2.3
4	NDUFAB1	3685306	-0.28	-2.9	-0.41	-4.6	-0.40	-5.2	-0.06	-0.3	0.03	-0.1	-0.29	-3.1	-0.31	-3.4
4	NDUFS1	2596201	-0.28	-2.9	-0.28	-2.3	-0.24	-2.1	0.10	-0.6	0.00	0.0	-0.17	-1.2	-0.17	-1.3
4	PINK1	2400210	-0.27	-2.7	-0.36	-3.8	-0.22	-1.8	0.12	-0.8	-0.33	-3.9	-0.13	-0.9	-0.12	-0.8
4	ATP5C1	3234140	-0.27	-2.7	-0.41	-4.6	-0.31	-3.4	0.15	-1.1	0.11	-0.7	-0.12	-0.7	0.05	-0.3
4	SLC25A23	3847873	-0.26	-2.5	-0.48	-6.5	-0.45	-6.6	0.04	-0.2	-0.27	-2.7	-0.40	-5.5	-0.06	-0.3
4	NDUFB6	3203162	-0.25	-2.3	-0.43	-5.1	-0.38	-4.8	0.15	-1.0	0.13	-0.9	-0.22	-1.8	-0.13	-0.8
4	COX15	3303109	-0.24	-2.2	-0.21	-1.4	-0.05	-0.3	0.08	-0.4	0.08	-0.5	-0.03	-0.1	-0.08	-0.5
4	SNCAIP	2826159	-0.24	-2.2	-0.41	-4.7	-0.17	-1.2	-0.48	-8.1	0.16	-1.2	-0.15	-1.1	-0.29	-3.1
4	GPD1	3414419	-0.24	-2.1	-0.38	-4.1	-0.43	-6.2	-0.21	-1.7	-0.38	-5.0	-0.36	-4.6	-0.30	-3.3

4	NDUFB9	3114649	-0.23	-2.1	-0.34	-3.3	-0.23	-1.9	0.05	-0.2	-0.22	-1.9	-0.22	-1.9	-0.25	-2.3
4	COX5A	3633221	-0.23	-2.1	-0.50	-6.9	-0.43	-6.2	0.08	-0.5	-0.32	-3.7	-0.36	-4.6	-0.35	-4.2
4	IGF1	3468345	-0.23	-2.0	-0.36	-3.6	-0.33	-3.7	0.14	-1.0	-0.41	-5.8	-0.38	-4.9	-0.37	-4.9
4	GPI	3829687	-0.22	-2.0	-0.22	-1.6	-0.25	-2.2	-0.27	-2.8	-0.25	-2.4	-0.22	-1.9	-0.26	-2.5
4	ENO2	3403015	-0.22	-2.0	-0.23	-1.7	-0.26	-2.4	-0.27	-2.7	-0.17	-1.3	-0.16	-1.1	-0.18	-1.4
4	NDUFS2	2363525	-0.22	-1.9	-0.17	-1.1	-0.12	-0.7	-0.02	-0.1	0.09	-0.5	-0.15	-1.1	-0.25	-2.4
4	INS	3359171	-0.22	-1.9	-0.06	-0.3	-0.26	-2.4	-0.07	-0.3	-0.02	-0.1	-0.02	-0.1	0.11	-0.7
4	HTR2A	3513147	-0.21	-1.8	-0.24	-1.8	-0.29	-2.8	0.23	-2.1	-0.31	-3.5	-0.25	-2.3	-0.32	-3.6
4	PFKFB4	2673312	-0.20	-1.7	-0.28	-2.3	-0.30	-3.0	0.01	0.0	-0.15	-1.0	-0.32	-3.7	-0.11	-0.6
4	PPP2R5D	2907538	-0.20	-1.6	-0.26	-2.0	-0.33	-3.6	-0.22	-2.0	-0.24	-2.2	-0.23	-2.1	-0.33	-3.8
4	COX10	3711165	-0.20	-1.6	-0.18	-1.2	-0.12	-0.7	-0.24	-2.2	-0.15	-1.0	-0.25	-2.4	-0.43	-6.4
4	NDUFC2	3341497	-0.20	-1.6	-0.14	-0.8	-0.40	-5.3	0.13	-0.8	-0.08	-0.4	-0.24	-2.1	0.03	-0.1
4	UQCRC2	3652218	-0.20	-1.6	-0.26	-2.0	-0.08	-0.4	-0.12	-0.7	0.08	-0.5	-0.06	-0.3	-0.20	-1.6
4	NDUFC1	2786578	-0.19	-1.5	-0.24	-1.8	-0.24	-2.1	0.38	-5.0	0.34	-4.1	0.09	-0.5	0.17	-1.2
4	PKLR	2437363	-0.19	-1.5	-0.21	-1.5	-0.38	-4.9	-0.17	-1.3	-0.16	-1.2	-0.29	-3.0	0.05	-0.2
4	NDUFB3	2522598	-0.18	-1.4	-0.23	-1.7	-0.32	-3.5	0.21	-1.8	-0.13	-0.9	-0.21	-1.7	-0.11	-0.6
4	GCK	3048468	-0.18	-1.4	-0.14	-0.8	-0.39	-5.1	0.08	-0.4	-0.12	-0.7	-0.21	-1.7	-0.02	-0.1
4	PRKAA2	2337716	-0.17	-1.2	-0.21	-1.5	-0.16	-1.1	0.11	-0.7	-0.15	-1.0	-0.12	-0.7	-0.12	-0.8
4	UQCR10	3942161	-0.16	-1.2	-0.38	-4.0	-0.39	-5.1	0.14	-0.9	0.00	0.0	-0.19	-1.5	-0.17	-1.3
4	DLD	3018696	-0.16	-1.1	-0.29	-2.4	-0.26	-2.4	0.02	-0.1	0.12	-0.7	-0.17	-1.2	-0.08	-0.5
4	NDUFA9	3401878	-0.15	-1.0	-0.30	-2.6	-0.25	-2.3	-0.05	-0.2	0.04	-0.2	-0.15	-1.1	-0.16	-1.1
4	COX4I1	3672455	-0.14	-1.0	-0.48	-6.3	-0.37	-4.5	0.17	-1.2	-0.09	-0.5	-0.01	0.0	0.03	-0.1
4	ACTN3	3336324	-0.14	-0.9	-0.17	-1.0	-0.19	-1.5	0.18	-1.4	-0.24	-2.3	-0.26	-2.5	0.04	-0.2
4	NDUFC2	3383138	-0.12	-0.8	-0.32	-3.0	-0.27	-2.6	0.01	-0.1	0.04	-0.2	-0.18	-1.4	-0.17	-1.3
4	ENO3	3707383	-0.12	-0.8	-0.21	-1.4	-0.20	-1.5	-0.15	-1.1	-0.11	-0.7	-0.15	-1.0	0.06	-0.3
4	GAPDHS	3830509	-0.12	-0.8	-0.13	-0.7	-0.22	-1.8	0.14	-1.0	-0.21	-1.8	-0.19	-1.5	-0.08	-0.5
4	NDUFS8	3337360	-0.12	-0.7	-0.26	-2.0	-0.23	-2.0	0.03	-0.1	-0.16	-1.2	-0.22	-1.9	-0.18	-1.4
4	SDHC	2363618	-0.10	-0.6	-0.44	-5.4	-0.15	-1.0	0.21	-1.7	-0.16	-1.2	-0.07	-0.4	-0.07	-0.4
4	NDUFS7	3815809	-0.10	-0.6	-0.23	-1.7	-0.20	-1.5	0.30	-3.3	0.13	-0.8	-0.19	-1.4	0.04	-0.2
4	HK2	2489545	-0.10	-0.6	-0.12	-0.6	0.12	-0.8	-0.01	0.0	-0.10	-0.6	-0.07	-0.3	-0.11	-0.7
4	SLC25A33	2319340	-0.08	-0.5	-0.09	-0.4	-0.11	-0.6	0.00	0.0	-0.16	-1.2	-0.13	-0.8	0.00	0.0
4	PGAM2	3048363	-0.08	-0.5	-0.19	-1.3	0.03	-0.1	-0.26	-2.5	-0.09	-0.5	0.03	-0.1	0.12	-0.7
4	NDUFB10	3644220	-0.08	-0.4	-0.44	-5.3	-0.23	-1.9	0.16	-1.1	-0.06	-0.3	-0.14	-1.0	-0.15	-1.0
4	FXN	3173831	-0.08	-0.4	-0.22	-1.6	-0.19	-1.5	0.18	-1.4	0.03	-0.1	-0.19	-1.5	-0.11	-0.7
4	MSH2	2480992	-0.06	-0.3	-0.29	-2.4	-0.18	-1.4	0.10	-0.6	0.06	-0.3	-0.09	-0.5	-0.15	-1.0
4	INSRR	2438612	-0.06	-0.3	0.17	-1.1	-0.31	-3.3	-0.24	-2.3	-0.07	-0.3	-0.08	-0.5	0.09	-0.5
4	TAZ	3996339	-0.05	-0.3	0.20	-1.4	0.36	-4.4	0.01	0.0	0.00	0.0	-0.01	0.0	-0.31	-3.4
4	NDUFS5	2331178	-0.05	-0.3	-0.31	-2.8	-0.27	-2.6	-0.19	-1.5	-0.27	-2.8	-0.21	-1.7	-0.33	-3.8
4	NDUFB5	2654069	-0.04	-0.2	-0.20	-1.3	-0.11	-0.6	-0.12	-0.7	-0.07	-0.4	-0.25	-2.3	-0.30	-3.3
4	GBAS	3003143	-0.04	-0.2	-0.14	-0.8	-0.27	-2.6	-0.11	-0.7	-0.23	-2.1	-0.17	-1.3	0.06	-0.3
4	MECP2	4027056	-0.04	-0.2	0.22	-1.6	0.23	-2.0	0.20	-1.7	0.34	-4.1	0.24	-2.2	-0.03	-0.2
4	MYOG	2451544	-0.04	-0.2	0.11	-0.6	-0.18	-1.3	-0.03	-0.1	0.06	-0.3	-0.04	-0.2	-0.02	-0.1
4	ALDOB	3218077	-0.04	-0.2	-0.32	-3.0	-0.26	-2.4	0.12	-0.8	0.00	0.0	-0.09	-0.5	-0.01	0.0
4	COX8A	3334125	-0.03	-0.2	-0.19	-1.3	0.00	0.0	-0.31	-3.5	-0.35	-4.4	-0.46	-7.4	-0.50	-8.9

4	MYC	3115504	-0.03	-0.1	-0.02	-0.1	-0.03	-0.1	-0.15	-1.1	-0.01	0.0	-0.13	-0.8	-0.06	-0.3
4	TPI1P2	3023279	-0.03	-0.1	-0.16	-1.0	-0.19	-1.5	0.00	0.0	-0.10	-0.6	-0.20	-1.7	-0.03	-0.1
4	NDUFV3	3922921	-0.02	-0.1	-0.12	-0.7	0.02	-0.1	-0.31	-3.4	-0.12	-0.7	-0.19	-1.5	-0.35	-4.3
4	PFKFB2	2377094	-0.01	-0.1	-0.16	-1.0	0.21	-1.8	0.15	-1.0	-0.09	-0.5	-0.05	-0.2	-0.21	-1.8
4	APOC3	3350655	-0.01	0.0	0.11	-0.6	-0.14	-0.9	0.12	-0.7	-0.12	-0.8	-0.11	-0.6	-0.06	-0.3
4	PRKAA1	2854737	-0.01	0.0	0.03	-0.1	0.25	-2.2	-0.20	-1.7	0.10	-0.6	-0.02	-0.1	-0.19	-1.5
4	NDUFA3	3841198	0.00	0.0	-0.11	-0.5	0.32	-3.5	0.01	0.0	-0.08	-0.4	-0.03	-0.1	-0.27	-2.7
4	ARNT	2434633	0.00	0.0	0.14	-0.8	0.33	-3.6	-0.09	-0.5	-0.02	-0.1	-0.03	-0.1	-0.24	-2.3
4	ATP7A	3982423	0.01	0.0	-0.08	-0.4	0.15	-1.0	0.24	-2.3	0.20	-1.6	-0.01	0.0	-0.13	-0.9
4	PMPCB	3017123	0.02	-0.1	0.12	-0.7	0.20	-1.6	0.13	-0.8	0.26	-2.5	0.14	-1.0	0.01	0.0
4	HK3	2888485	0.02	-0.1	0.13	-0.7	-0.33	-3.6	-0.15	-1.1	-0.04	-0.2	0.00	0.0	0.07	-0.4
4	NDUFV2	3778207	0.04	-0.2	-0.11	-0.6	-0.10	-0.5	0.13	-0.9	0.04	-0.2	-0.10	-0.6	0.08	-0.4
4	SDHAF2	3332938	0.04	-0.2	-0.27	-2.3	0.09	-0.5	0.18	-1.4	0.22	-1.9	0.11	-0.6	-0.02	-0.1
4	HIF1A	3539070	0.06	-0.3	0.04	-0.2	0.21	-1.7	0.07	-0.4	0.21	-1.8	0.12	-0.8	0.09	-0.5
4	PPIF	3253880	0.06	-0.3	-0.17	-1.1	0.04	-0.2	0.41	-5.9	0.00	0.0	-0.15	-1.1	-0.19	-1.5
4	NDUFA10	2606574	0.07	-0.3	0.10	-0.5	0.28	-2.8	-0.05	-0.3	0.21	-1.8	0.22	-1.8	-0.10	-0.6
4	MLXIPL	3056163	0.07	-0.4	-0.01	0.0	-0.16	-1.1	-0.01	0.0	-0.33	-4.0	-0.15	-1.1	-0.29	-3.2
4	COX4I2	3881404	0.08	-0.4	0.08	-0.4	-0.08	-0.4	0.10	-0.6	-0.03	-0.1	-0.01	0.0	-0.04	-0.2
4	UQCRB	3145564	0.08	-0.4	-0.18	-1.1	-0.13	-0.8	0.26	-2.5	0.18	-1.3	0.01	0.0	-0.02	-0.1
4	NDUFS4	2809423	0.09	-0.5	-0.34	-3.3	-0.19	-1.4	0.01	0.0	0.04	-0.2	-0.17	-1.2	-0.16	-1.1
4	PFKFB1	4009811	0.12	-0.7	0.15	-0.8	0.35	-4.1	0.19	-1.5	0.21	-1.8	0.06	-0.3	0.15	-1.0
4	PFKM	3452970	0.15	-1.0	0.18	-1.1	0.31	-3.3	-0.12	-0.8	0.04	-0.2	0.05	-0.3	-0.12	-0.8
4	COX8C	3549381	0.17	-1.2	0.07	-0.3	0.31	-3.3	0.40	-5.7	0.17	-1.2	0.26	-2.5	0.16	-1.2
4	DNAJC15	3487432	0.19	-1.5	-0.25	-1.9	0.07	-0.4	0.05	-0.3	-0.12	-0.7	-0.05	-0.2	-0.16	-1.2
4	COQ9	3662723	0.20	-1.7	0.26	-2.1	0.32	-3.5	0.11	-0.7	0.19	-1.5	0.20	-1.6	-0.04	-0.2
4	P2RX7	3434726	0.21	-1.7	0.24	-1.8	0.41	-5.7	0.48	-8.0	0.03	-0.2	0.17	-1.3	0.12	-0.8
4	AK2	2405364	0.21	-1.8	0.14	-0.8	0.32	-3.4	-0.20	-1.6	0.13	-0.8	0.08	-0.4	-0.11	-0.7
4	NDUFA2	2878446	0.23	-2.1	-0.32	-2.9	-0.06	-0.3	0.07	-0.4	0.06	-0.3	0.09	-0.5	-0.14	-1.0
5	CRH	3138618	-0.27	-2.6	-0.15	-0.9	-0.39	-5.0	0.19	-1.5	-0.22	-1.9	-0.57	-11	-0.43	-6.3
5	NR3C1	2879312	-0.23	-2.1	-0.05	-0.2	-0.05	-0.2	-0.19	-1.5	-0.02	-0.1	-0.17	-1.3	-0.30	-3.3
5	RXRA	3193339	-0.13	-0.8	0.00	0.0	-0.22	-1.8	0.61	-13	0.17	-1.3	0.13	-0.9	0.29	-3.0
5	PPARG	2611056	-0.09	-0.5	-0.11	-0.6	-0.17	-1.2	-0.17	-1.3	-0.11	-0.7	-0.01	0.0	0.05	-0.3
5	NR3C2	2788926	-0.08	-0.4	-0.22	-1.6	-0.17	-1.2	0.38	-5.0	-0.17	-1.2	0.06	-0.3	-0.12	-0.8
5	PPARD	2904597	0.02	-0.1	0.25	-1.9	-0.08	-0.4	0.19	-1.6	0.08	-0.5	-0.30	-3.2	-0.44	-6.8
5	PPARA	3948953	0.02	-0.1	0.14	-0.8	0.31	-3.3	0.58	-12	0.37	-4.9	0.31	-3.4	0.40	-5.6
5	GPR37	3070873	0.04	-0.2	0.12	-0.6	0.19	-1.5	-0.01	0.0	0.06	-0.3	-0.03	-0.1	0.12	-0.8
5	NPY	2993124	0.06	-0.3	0.05	-0.2	-0.34	-4.0	-0.06	-0.3	-0.42	-6.1	-0.31	-3.4	-0.28	-2.8
5	HSPA2	3540136	0.14	-0.9	0.09	-0.4	0.29	-2.9	0.22	-1.9	0.20	-1.6	0.08	-0.5	0.25	-2.4
5	FKBP5	2951567	0.45	-7.0	0.39	-4.2	0.51	-8.8	0.59	-13	0.57	-11	0.60	-13	0.57	-11

Group: 1) Telomere length inheritance (other than group 2 and 3), 2) Telomerase biogenesis, 3) Telomere maintenance, 4) Oxidative phosphorylation, 5) Stress / neuroprotection
Probe: DNA array probe ID for GPL5175

PUTM: putamen, SNIG: substantia nigra, HIPP: hippocampus, CRBL: cerebellar cortex, FCTX: frontal cortex, OCTX: occipital cortex (specifically primary visual cortex), WHMT: intralobular white matter

r: Pearson's correlation coefficient (Red: positive correlation, Blue: negative correlation)
p: p-value for Student t-distribution expressed as log10 (Darker brown: greater significance)
